# Anticancer Plant Secondary Metabolites Induce Linker Histone Depletion from Chromatin

**DOI:** 10.31083/j.fbl2908275

**Published:** 2024-08-05

**Authors:** Olga Vlasova, Irina Antonova, Roman Zenkov, Denis Naberezhnov, Gennady Belitsky, Anna Borunova, Tatiana Zabotina, Daniel García-Gomis, Alfiya Safina, Katerina Gurova, Andrei Gudkov, Kirill Kirsanov, Albert Jordan, Marianna Yakubovskaya

**Affiliations:** 1 N.N. Blokhin National Medical Research Center of Oncology, Ministry of Health of Russia, 115478 Moscow, Russia; 2 Federal State Budgetary Institution, Centre for Strategic Planning and Management of Biomedical Health Risks of the Federal Medical and Biological Agency, 119121 Moscow, Russia; 3 Engelgardt Institute of Molecular Biology, Russian Academy of Sciences, 119991 Moscow, Russia; 4 Department of Molecular Genomics, Molecular Biology Institute of Barcelona IBMB-CSIC, Scientific Park of Barcelona, 08028 Barcelona, Catalonia, Spain; 5 Department of Cell Stress Biology, Roswell Park Comprehensive Cancer Center, Buffalo, NY 14263, USA; 6 Department of Faculty Surgery, Peoples’ Friendship University of Russia Miklukho-Maklaya St., 117198 Moscow, Russia

**Keywords:** plant secondary metabolites, chromatin structure, linker histones, cancer preventive activity, cancer prevention, DNA-binding compounds, cell cycle, cytotoxicity, nucleosome stability, linker histone depletion

## Abstract

**Background:**

Many plant secondary metabolites (PSMs) were shown to intercalate into DNA helix or interact with DNA grooves. This may influence histone-DNA interactions changeing chromatin structure and genome functioning.

**Methods:**

Nucleosome stability and linker histone H1.2, H1.4 and H1.5 localizations were studied in HeLa cells after the treatment with 15 PSMs, which are DNA-binders and possess anticancer activity according to published data. Chromatin remodeler CBL0137 was used as a control. Effects of PSMs were studied using fluorescent microscopy, flowcytometry, quantitative reverse transcriptase-polymerase chain reaction (RT-qPCR), western-blotting.

**Results:**

We showed that 1-hour treatment with CBL0137 strongly inhibited DNA synthesis and caused intensive linker histone depletion consistent with nucleosome destabilization. None of PSMs caused nucleosome destabilization, while most of them demonstrated significant influence on linker histone localizations. In particular, cell treatment with 11 PSMs at non-toxic concentrations induced significant translocation of the histone H1.5 to nucleoli and most of PSMs caused depletion of the histones H1.2 and H1.4 from chromatin fraction. Curcumin, resveratrol, berberine, naringenin, and quercetin caused significant redistribution of all three variants of the studied linker histones showing some overlap of PSM effects on linker histone DNA-binding. We demonstrated that PSMs, which induced the most significant redistribution of the histone H1.5 (berberine, curcumin and naringenin), influence the proportion of cells synthesizing DNA, expressing or non-expressing cyclin B and influence cell cycle distribution. Berberine induction of H1.5 translocations to nucleoli was shown to occur independently on the phases of cell cycle (metaphase was not analyzed).

**Conclusions:**

For the first time we revealed PSM influence on linker histone location in cell nuclei that opens a new direction of PSM research as anticancer agents.

## 1. Introduction

A series of significant studies clearly demonstrated that the Mediterranean diet may slow down the incidence of various forms of cancer [1]. This effect is proposed to be due at least partly to high concentrations of plant secondary metabolites (PSMs) from the group of polyphenols. These compounds are characterized by the presence of one or more aromatic rings with different hydroxyl groups, which determine their chemical properties and biological effects. A large number of cellular targets for these small molecules have been found, as well as mediated changes in specific signaling pathways that prevent tumor growth or carcinogenesis. Many studies have demonstrated antiproliferative, proapoptotic, anti-inflammatory, and immunomodulatory effects of resveratrol, quercetin, genistein and other PSMs in the experiments *in vitro* [2–5]. In the experiments *in vivo*, a number of PSMs were demonstrated to reduce the incidence and multiplicity of colon tumors induced by 1,2-dimethylhydrazine or azoxymethane, ovarian tumors induced by 7,12-dimethylbenz(a)anthracene, and mammary tumors induced by 7,12-dimethylbenz(a)anthracene and N-methyl-N-nitrosourea [6–9]. Their chemopreventive effects was shown in a number of clinical trials [10–12].

The pleiotropy of the effects of PSMs makes it extremely difficult to interpret the integral result of their action. PSMs are able to bind to a large number of protein molecular targets, including cellular receptors, enzymes of xenobiotic metabolism, components of signaling pathways, enzymes of DNA metabolism and epigenetic regulation of transcription [13–16]. However, one of the commonest properties of PSMs is their affinity to DNA.

Noteworthy, PSMs do not form covalent bonds with DNA, but interact with the biopolymer via van der Waals, ionic, and hydrogen bonds. This explains why PSMs are mainly not mutagenic. Using various spectroscopic methods, PSM intercalation into DNA helix was demonstrated for resveratrol and genistein [17], quercetin and delphinidin [18], apigenin and naringenin [19], fisetin [20], epigallocatechin gallate (EGCG) [21], and sanguinarine [22]. DNA minor groove binding was shown for curcumin [17] and sanguinarine [23]. Binding DNA, PSMs can affect the geometric characteristics and thermodynamic stability of the duplex, the flexibility and physicochemical properties of the biopolymer, as well as influence the formation and stabilization of various alternative DNA structures, such as G-quadruplexes, H-DNA, and cruciforms. Binding and stabilization of G-quadruplexes by PSMs were shown for fisetin [20], curcumin [24], EGCG [25], kaempferol [26], berberine [27], sanguinarine [28], quercetin [29], and thymoquinone [30]. G4-mediated downregulation of c-MYC expression was shown by reporter analysis for sanguinarine, quercetin, kaempferol and thymoquinone [31]. Moreover, they can shield the sites of DNA interaction with enzymes that are involved in replication, transcription, and repair. All the DNA-mediated effects of PSMs may influence the three-dimensional organization of chromatin and DNA packaging processes and change the access to DNA landing sites for chaperones, epigenetic factors, and other regulatory proteins [32,33]. In our previous studies, the effects of chromatin destabilization by DNA-binding agents have been described for the new anticancer and chemopreventive drug Curaxin CBL0137 [34,35] and for minor groove binding ligands (MGBL) [36]. CBL0137 does not affect the chemical structure of DNA, but it causes dose-dependent destabilization and disassembly of the nucleosome [35], followed by histone chaperone FACT trapping to chromatin [37] and cell cycle arrest at G0/G1 and G2/M phases.

Influence of PSMs on cell chromatin structure has never been analyzed. The main goals of our study were to analyze PSM effects on chromatin stability, nucleosome histones and histone chaperone facilitates chromatin transcription (FACT) localization in the cell nuclei, linker histones localization in the nuclei and their relative content in chromatin fraction and total protein pool, cell cycle distribution under the treatment of PSMs and the dependence of PSM effects on cell cycle phase. 15 PSMs of different chemical structures, which demonstrate DNA-binding and possess anticancer activity, were chosen for our study.

## 2. Materials and Methods

### 2.1 Chemicals and Reagents

All of the studied compounds were obtained from Chemlight, Moscow, Russia (1) and from Selleckchem, Houston, TX, USA (2). We studied apigenin, CAS 520–36-5, Lot. 0436771–86, (1); berberine, CAS 633–65-8, Lot. S904601, (1); coumarin, CAS 91–64-5, (1); curcumin, CAS 458–37-7, (1); delphinidin, CAS 13270–61-6, Lot. 117271994, (1); EGCG, CAS 989–51-5, Lot. SLBL3516V, (1); fisetin, CAS 528–48-3, Lot. 477164536, (1); genistein, CAS 446–72-0, Lot. S134201, (1); ginsenoside, Rb1, CAS 41753–43-9, Lot S392401, (1); kaempferol, CAS 520–18-3, Lot. S231406, (1); naringenin, CAS 480–41-1, (1); quercetin, CAS 117–39-5, Lot. LRAB3054, (1); resveratrol, CAS 501–36-0, Lot. 139609, (1); sanguinarine chloride hydrat, CAS 5578–73-4, Lot. S903202, (1); thymoquinone, CAS 490–91-5, Lot. S476101, (1). Curaxin CBL0137 was provided by Incuron, Inc., Russia. Triton X-100 (CAS 9002–93-1, Lot. 19H2156267) was purchased from BioInnlabs, Rostov-on-Don, Russia. Phenol – chloroform – isoamyl alcohol mixture (CAS 136112–00-0), TopVision Low Melting Point Agarose (CAS 39346–81-1, Lot. 91339087), cOmplete^™^, Mini Protease Inhibitor Cocktail (cat. 11836153001, Lot. 33576300), Laemmli Sample Buffer x2 (CAS 53123–88-9, Lot. S3401), Phosphate-buffered saline (PBS, P4417, Lot. M8254), DAPI (CAS 28718–90-3, Lot. 378275275), thymidine (50–89-5, Lot. WXBD3213V), 5-Ethynyl-2′-deoxyuridine (EdU, 61135–33-9, Lot. 2161886), 4% paraformaldehyde (CAS 30525–89-4), bovine serum albumin (CAS 9048–46-8), Resazurin sodium salt (CAS 62758–13-8, Lot. MKCG9800) and G418 disulfate salt solution (CAS 108321–42-2) were purchased from Sigma Aldrich (Merck), Bengaluru, Karnataka, India. Micrococcal nuclease derived from Staphylococcus aureus (CAS 9013–53-0, Lot. 00473368), Q5^®^ High-Fidelity DNA Polymerase (M0491L, Lot. 509DR50200), T4 Polynucleotide Kinase (M0201L) and T4 DNA ligase (M0202T) were obtained from NEB, USA. DC^™^ Protein Assay Kit I (5000111EDU) was purchased from Bio-Rad (Moscow, Russia). Clarity Max^™^ Western ECL Substrate for Chemiluminescent Detection of Horseradish Peroxidase (HPR) Conjugates (cat. 1705062, Lot. 102031834) was purchased from Helicon, Moscow, Russia. Click-iT^™^ EdU Cell Proliferation Kit (C10337, Lot. 2161886), TRIzol^™^ Reagent (15596026, Lot. 03211240170), TurboFect Transfection Reagent (R0531), M-MLV Reverse Transcriptase Buffer (18057018, Lot. EA029021173) and FastAP alkaline phosphatase (EF0651) were purchased from Thermo Scientific (Invitrogen), Waltham, MA, USA. Nuclear Marker and ChIP Grade (ab1791, Lot. 00211074) was purchased from Abcam (Cambridge, UK). Hexanucleotides (6-random, Random Hexa Primer, D0300) was purchased from Sileks (Moscow, Russia). 2.5x Reaction mixture for quantitative reverse transcriptase-polymerase chain reaction (RT-qPCR) in the presence of SYBR Green I dye (M-427, Lot. SA001021272) was purchased from Syntol (Moscow, Russia). Plasmid pCMV(CAT)T7-SB100 (34879) was obtained from Addgene (Watertown, MA, USA).

### 2.2 Cell Cultures

Human cervical cancer cells (HeLa) was obtained from the Blokhin Cancer Research Center (CRC) cell collection, HeLa_SSRP1-GFP_H2B-mCherry were obtained from the Department of Cell Stress Biology at Roswell Park (Buffalo, NY, USA). HeLa_H1.5-mCherry cells were constructed as described below. Cells were cultured in Dulbecco’s Modified Eagle Medium (DMEM) supplemented with L-glutamine (0.584 mg/mL), penicillin (50 U/mL), and streptomycin (50 μg/mL) (PanEco, Moscow, Russia) and 10% fetal bovine serum (PanEco). Cell lines were incubated at 37 °C and 5% CO_2_. All cell lines were validated by STR profiling and tested negative for mycoplasma.

### 2.3 Constructing HeLa-H1.5-mCherry Cell Line

Vector pSB/IR-TRE3G-H1-mCherry was constructed in order to provide expression of fluorescent linker histone H1.5 in HeLa cells. This plasmid includes the gene of H1-mCherry fusion protein and inverted repeats that are crucial for transposition (via “sleeping beauty” mechanism).

The sequence of H1-mCherry gene and the gene of resistance to geneticin was amplified from pReceiver-M55 plasmid provided by the laboratory of Katerina Gurova. Amplification was carried out with Q5 polymerase (NEB, Ipswich, MA, USA) and the following primers:

forward: 5′-GAGATATCTAAGATACATTGATGAGTTTGGAC-3′

reverse: 5′-CGCCACCATGGTGAGCAAGGGCG-3′

Blunt ends of the product were kinated with T4 Polynucleotide Kinase (NEB, Ipswich, MA, USA).

Plasmid pSB-IR-TRE3G-5.1, which contains inverted repeats for transposition and TRE3G promotor (with the tetracycline controlled transcriptional activation), was hydrolyzed with EvoRV restrictase resulting in DNA fragment with blunt ends. Linear DNA fragment was incubated with FastAP alkaline phosphatase (ThermoScientific, Waltham, MA, USA) in order to avoid circularization of the fragment during ligation.

Two fragments were ligated with T4 DNA ligase (SibEnzyme, Novosibirsk, Russia). Bacterial colonies with the proper construction were selected in PCR with the following primers:

forward: 5′-CTGAGCTAGCTTACTTGTACAGCTCGTCC-3′

reverse: 5′- CGATTTTTGTGATGCTCGTCAGG-3′

PCR with proper construction resulted in the DNA product of 1773 bp. Plasmid sequence was confirmed by sequencing.

Final plasmid pSB/IR-TRE3G-H1-mCherry was transfected into HeLa cells in 60 mm dishes using TurboFect Transfection Reagent (ThermoScientific, Waltham, MA, USA) according to the manufacturer’s protocol. It was transfected with plasmid pCMV(CAT)T7-SB100 (Addgene, 34879) in the ratio of 4 to 1. pCMV(CAT)T7-SB100 encodes sleeping beauty SB100X transposase, which inserts the construction into cell genome. Cells containing the construction in their genome were selected with geneticin (G418).

### 2.4 Cell Viability Assay

The nontoxic concentrations of phytonutrients for Hela cells were measured using the resazurin test based on the fluorescent determination of the metabolic activity of viable cells. For analysis, HeLa cancer cells were seeded in 96-well plates (10,000 cells per well in 180 μL of DMEM). Cells were treated with phytonutrients of various concentrations for 24 hours. Then, 20 μL of resazurin reagent solution (0.18 mg/mL) was added to each well and incubated for 4 h at 37 °C. The plates were read on a Fluoroscan FL (Thermo Scientific, Waltham, MA, USA) using a test wavelength of 530 nm. IC50 and IC20 values and nontoxic concentrations were derived from aproximate logistic function of resorufin fluorescence intensity at 592 nm. Maximum non-toxic concentrations were confirmed using propidium iodide (PI)/annexin cell staining and subsequent flow cytometric analysis. All experiments were carried out in parallel and in triplicate.

### 2.5 Micrococcal Nuclease Digestion Assay

Micrococcal nuclease digestion assay was performed as previously described [35] with some modifications. HeLa cells were trypsinized, harvested, and washed once with 1× RSB buffer (10 mM Tris, pH7.6, 15 mM NaCl and 1.5 mM MgCl_2_). After centrifugation, the cell pellet was resuspended in 1× RSB buffer with 1% Triton X-100 and homogenized by five strokes with a loose-fitting pestle to release nuclei. Nuclei were collected by centrifugation (2000 g, 5 min) and washed twice with 1 mL of fresh buffer A (15 mM Tris, pH 7.5, 15 mM NaCl, 60 mM KCl, 0.34 M sucrose, 0.5 mM spermidine, 0.15 mM spermine, 0.25 mM PMSF and 0.1%-mercaptoethanol). Finally, nuclei (from 20 × 106 cells) were resuspended in 1.5 mL of buffer A, and 15 μL of 0.1 M CaCl_2_ was added. Concentrated solutions of all analyzed compounds were prepared in dimethyl sulfoxide (DMSO) or ethanol, and the concentration of solvents in the incubation medium of cell nuclei after the addition of compounds was 0.4%, which had no significant effect on either the epigenetic regulation of transcription or transcription activity in HeLa TI cells. HeLa cell nuclei were treated at first with CBL0137 or PSMs for 30 min at 37 °C and then with NEB micrococcal endonuclease for 15 min. To control the quality of the untreated chromatin used in the experiment, nuclease digestion was stopped immediately after the enzyme addition to the nuclei untreated with PSMs. For digestion, 1 μL of 200 U/mL micrococcal nuclease (NEB, Ipswich, MA, USA) was added to 0.5 mL nuclei suspension at 37 °C. Aliquots (60 μL) were taken after 15 min, and 1.5 μL 0.5 M EDTA was added to stop the reaction (final concentration: 12.5 mM). Subsequently, 18 μL H_2_O, 12 μL of 10% SDS and 24 μL of 5 M NaCl were added to each tube. The mixtures were extracted with phenol–chloroform followed by ethanol-based precipitation. DNA was analyzed on a 1.5% agarose gel, stained with 0.5 g/mL ethidium bromide in 1× TAE for 30 min, destained for 2 × 15 min in ddH_2_O, and imaged as described above.

### 2.6 Cell Microscopy

To visualize the localization of core histone H2B and linker histone H1.5 cells HeLa_SSRP1-GFP_H2B-mCherry or HeLa_H1.5-mCherry were seeded in 6-well plates (150,000 cells per well) with cover glass 22 × 22 mm for microscopy. After 24 hours, cells were incubated with CBL0137 (positive control) for 1 hour or with phytonutrients for 1 hour or 24 hours. After the treatment, cells were washed twice with PBS and fixed in 4% paraformaldehyde at room temperature for 15 min. After three washes, the nuclei were stained with DAPI. The coverslips were mounted on the glass slides using Mowiol mounting medium. Images were obtained with a Zeiss Axio Observer A1 inverted microscope (Carl Zeiss, Munich, Germany) with N-Achroplan 100×/1.25 oil lens (Carl Zeiss, Munich, Germany), Zeiss MRC5 camera (Carl Zeiss, Munich, Germany), and AxioVision Rel.4.8 software (Carl Zeiss Microscopy, LLC, White Plains, NY, USA). Experiments were performed three times. 200 cells were analized for each point. Image analyses was done using ImageJ (version 1.48; National Institute of Health, Bethesda, MD, USA).

### 2.7 Cell Fractionation for Chromatin and Nucleoplasmic Proteins Extraction, Gel Electrophoresis, and Immunoblotting

For isolation of total protein, cells were incubated with RIPA buffer (50 mM Tris–HCl, pH 7.4, 1% NP-40, 0.5% Na-deoxycholate, 450 mM NaCl, 1 mM EDTA, 1 mM EGTA), with Complete Protease Inhibitor Cocktail (Roche, Basel, Switzerland) on ice for 30 min. For chromatin and nucleoplasmic fraction extraction 10 million cells were resuspended in 1 mL of Buffer A (10 mM HEPES-KOH, pH 7.9, 1.5 mM MgCl_2_, 10 mM KCl, 0.5 mM DTT) with Complete Protease Inhibitor Cocktail (Roche, Basel, Switzerland) and incubated for 10 min on ice. Then the cell pellet was homogenized by 50 strokes with a loose-fitting pestle. The homogenized suspension was centrifuged at 600 g for 5 min at 4 °C. The remaining pellet was resuspended in High Salt Buffer-1 (20 mM HEPES-KOH, pH 7.9, 25% glycerol, 1.5 mM MgCl_2_, 0.42 M NaCl, 0.2 mM EDTA) with Complete Protease Inhibitor Cocktail (Roche, Basel, Switzerland) and was incubated 90 min at 4 °C gently mixing on a shaker. Then it was centrifuged at 16,000 g for 30 min at 4 °C and the supernatant was taken as the nucleoplasmic fraction. The pellet was resuspended in High Salt Buffer-2 (20 mM HEPES-KOH, pH 7.9, 25% glycerol, 1.5 mM MgCl_2_, 0.75 M NaCl, 0.2 mM EDTA) with Complete Protease Inhibitor Cocktail (Roche, Basel, Switzerland) and boiled 5 min at 95 °C. Then it was centrifuged at maximum velocity for 15 min at 4 °C and the supernatant was collected as the chromatin-bound fraction of proteins [38].

Protein concentrations were determined with the Bio-Rad DC protein assay kit (Hercules, CA, USA). Samples were mixed with Laemmli Gel Loading buffer and boiled for 5 min at 95 °C.

Samples were exposed to polyacrylamide gel with sodium dodecyl sulfate (SDS-PAGE) (10%), transferred to a Polyvinylidene fluoride (PVDF) membrane, blocked with 5% non-fat milk for 1 hour, incubated with primary antibodies over night at 4 °C and secondary antibodies conjugated to peroxidase for 1 hour at room temperature. Bands were detected using Clarity Western substrate for enhanced chemiluminescence (ECL) visualization reagent (1705061, Bio-Rad, Hercules, CA, USA) and ImageQuant LAS 4000 digital imaging system (GE Healthcare, Chicago, IL, USA). Densitometric analysis of the blots was performed using ImageJ software. Experiments were performed minimum three times.

We used the following pimary antibodies: SSRP1, mouse monoclonal 10D1 (BioLegend, cat# 609702, San Diego, CA, USA); SPT16, mouse monoclonal 8D2 (BioLegend, cat# 607002, San Diego, CA, USA); β-actin (Santa Cruz Biotechnology cat# sc-47778; 1:20,000, Dallas, TX, USA); H3 (abcam cat# 1791, Cambridge, UK); H1.2 (abcam cat#17677, Cambridge, UK); H1.4 (abcam cat#105522, Cambridge, UK); PARP1 (Cell Signaling cat#9532S, Danvers, MA, USA) and secondary antibodies: mIG-HRP (Santa Cruz Biotechnology cat#sc-516102, Dallas, TX, USA).

### 2.8 Quantitative Reverse Transcriptase-Polymerase Chain Reaction (RT-qPCR)

The effect of phytonutrients on H1 genes expression was evaluated in cell line HeLa using the RT-qPCR assay. Cells were incubated for 24 h in the medium with various phytonutrients using concentration IC20 in 6-well plates. Then, total RNA was extracted with TRIzol reagent (ThermoScientific, Waltham, MA, USA) according to the manufacturer’s protocol. For cDNA synthesis, total RNA (1 μg from both untreated and phytonutrient-treated cells) was reverse transcribed using Moloney Murine Leukemia Virus Reverse Transcriptase (MMLV-RT) reverse transcriptase and random hexanucleotide primers in 25 μL of the reaction mixture according to the manufacturer’s protocol (Syntol). Each PCR was based on 5 ng of DNA, 1× PCR Buffer, 0.3 mM dNTPs, 3 mM MgCl_2_, 0.2 U Syn Taq DNA polymerase, and 0.2 μM forward and reverse primers in a 25-μL reaction volume. RT-qPCR was carried out with a CFX96 Touch^™^ Real-Time PCR detection system from Bio-Rad Laboratories (Moscow, Russia). Real-time RT-qPCR was performed using the following thermal cycling conditions: initial denaturation step by heating at 95 °C for 5 min, followed by 40 cycles of 15 s denaturation (at 95 °C), 20 s at the appropriate temperature (depending on the Tm values of the primer used) and 25 s of extension at 72 °C. Expression of the gene of interest was normalized to the constitutively expressed housekeeping gene *RPL27*. The relative expression level was calculated for each sample using the 2^*−*∆∆Ct^ method.

The sequences of the gene-specific primers used for RT-qPCR were as follows:

- RPL27 F: 5′ACCGTCACCCCCGCAAAGTG 3′
- RPL27 R: 5′CCCGTCGGGCCTTGCGTTTA 3′
- H1–0 F: 5′CCTGCGGCCAAGCCCAAGCG 3′
- H1–0 R: 5′AACTTGATCTGCGAGTCAGC 3′
- H1–1 F: 5′CTCCTCTAAGGAGCGTGGTG 3′
- H1–1 R: 5′GAGGACGCCTTCTTGTTGAG 3′
- H1–2 F: 5′GGCTGGGGGTACGCCT 3′
- H1–2 R: 5′TTAGGTTTGGTTCCGCCC 3′
- H1–3 F: 5′CTGCTCCACTTGCTCCTACC 3′
- H1–3 R: 5′GCAAGCGCTTTCTTAAGC 3′
- H1–4 F: 5′GTCGGGTTCCTTCAAACTCA 3′
- H1–4 R: 5′CTTCTTCGCCTTCTTTGGG 3′
- H1–5 F: 5′CATTAAGCTGGGCCTCAAGA 3′
- H1–5 R: 5′TCACTGCCTTTTTCGCCCC 3′

### 2.9 Analysis of Cell Cycle Phase Distribution and Monitoring of Cell Cycle Synchronization Using Flow Cytometry

For Analysis of cell cycle phase distribution HeLa_H1.5-mCherry was seeded in 6-well plates (100,000 cells per well). After 24 hours, cells were treated with compounds of interest at IC20 concentrations for 1 hour. Next, the cells were incubated for 20 min with EdU (20 μM) and removed from the substrate with trypsin.

For monitoring of cell cycle synchronization HeLa_H1.5-mCherry was seeded in 6-well plates (50,000 cells per well). After 24 hours to synchronize cells in S phase, cells were treated with thymidine (final concentration 2 μM), incubated for 14–16 hours, then cells were washed twice with PBS, culture medium was added and incubated for 6–10 hours. Cells were then washed twice with PBS and treated again with thymidine. Cells were incubated for 20 min with EdU (20 μM) and removed from the substrate with trypsin: for S-phase fixation after 15 hours, for G2/M-phase fixation after 21 hours, for G1-phase fixation after 29 hours.

After the above treatments, cell lines were washed several times with PBS and fixed in cold 4% paraformaldehyde for 15 min. After three washes, they were permeabilized with cold Triton-X100 for 7 min and blocked with bovine serum albumin for 1 hour. Then S-phase labeling was carried out in a fast, highly-specific click reaction (Invitrogen^™^Click-iT^™^ EdU Cell Proliferation Kit, Waltham, MA, USA). To mark the G2/M phase, immunofluorescent staining of cells with antibodies to cyclin B1 (Invitrogen cat#MA5–13128, Waltham, MA, USA) and subsequent binding with secondary antibodies AlexaFluor647 (Abcam cat#150115, Cambridge, UK) were performed in darkness. Cells were washed with PBS and analyzed on a FACS Canto-2 flow cytometer. Thus, a cell in S-phase had a phenotype (EdU+, cyclin B1–), and in a cell in G2/M-phase it had a phenotype (EdU–, cyclin B1+). The absence of both markers (EdU–, cyclin B1–) is a sign of cell identification in the G1/G0 phase.

Also cell cycle analysis was carried out according to PI staining protocol (Invitrogen, Waltham, MA, USA). Cells were collected, washed twice with ice-cold PBS and fixed with 70% ethanol at 4 °C 30 min. Then cells were stored at –20 °C for 24 h, then washed with PBS and stained with 0.02 mg/mL PI, 0.1% v/v Triton X-100 and 0.2 mg/mL DNase-free RNase A in PBS. After 30 min incubation at room temperature in the dark, cells were analyzed by flow cytometer FACS Canto-2. Percent of cells in each cell cycle phase was analyzed and calculated using kaluza software, version 2.2.1 (https://www.beckman.co.il/ru/flow-cytometry/software/kaluza).

### 2.10 Synchronization, Immunostaining and Microscopy

Synchronization of the cell cycle of the HeLa H1.5-mCherry line was carried out using a double thymidine block with subsequent analysis of the distribution of the cell population over the phases of the cell cycle at different time points after the removal of the thymidine block by analyzing immunofluorescently stained cells on a flow cytometer.

HeLa_H1.5-mCherry was seeded in 6-well plates (50,000 cells per well) with a 22 × 22 mm coverslip for microscopy. After 24 hours, to synchronize the cells in the S phase, the cells were treated with thymidine (final concentration 2 μM), incubated for 14–16 hours, then the cells were washed twice with PBS, culture medium was added, and incubated for 6–10 hours. Cells were then washed twice with PBS and treated again with thymidine. Cells were treated with compounds of interest for 1 hour and then incubated for 20 minutes with EdU (20 μM): for S-phase fixation after 15 hours, for G2/M-phase fixation after 21 hours, for G1-phase fixation after 29 hours.

To identify cells in certain cell phases after the above cell line treatments cells were washed twice with PBS and fixed in 4% paraformaldehyde at room temperature for 15 min. After three washes, they were permeabilized with Triton-100 for 7 min at room temperature and blocked with bovine serum albumin for 1 h. Then S-phase labeling was carried out in a fast, highly-specific click reaction (Invitrogen^™^Click-iT^™^ EdU Cell Proliferation Kit, Waltham, MA, USA). To mark the G2/M phase, immunofluorescent staining of cells with antibodies to cyclin B1 (Invitrogen cat#MA5–13128, Waltham, MA, USA) and subsequent binding with secondary antibodies AlexaFluor647 (Abcam cat#150115, Cambridge, UK) were performed in darkness. Thus, cells in S-phase had a phenotype (EdU+, cyclin B1–), cells in G2/M-phase had a phenotype (EdU–, cyclin B1+). The absence of both markers (EdU–, cyclin B1–) made it possible to identify cells in the G1/G0 phase. The nucleus was stained with DAPI. The coverslips were mounted on the glass slides using Mowiol mounting medium. Images were obtained with a Zeiss Axio Observer A1 inverted microscope with N-Achroplan 100×/1.25 oil lens, Zeiss MRC5 camera, and AxioVision Rel.4.8 software. Experiments were performed three times. 200 cells were analized for each point. Image analyses and quantitification was done using ImageJ.

### 2.11 Statistical Analysis

We compared data of the treatments and controls using one-way analysis of variance (ANOVA) and Dunnett’s post hoc test. Differences between groups were considered to be significant at a *p* value of <0.05. Statistically significance of the differences of the histone contents in nucleoli and intermediate position in cell nucleos was by Pearson’s chi-squared test. Statistical analyses were performed using GraphPad Prism 8.3.0 (GraphPad Software Inc., San Diego, CA, USA).

## 3. Results

### 3.1 HeLa Cell Viability under the Treatment of Plant Secondary Metabolites

We wanted to use non-toxic concentration of PSMs, and before studying the effects of PSMs on the chromatin structure we measured their cytotoxicity using resazurin assay. IC50 and IC20 were determined from the fit curves to dose-response data (Table 1, Supplementary Fig. 1). IC0 was determined as a maximal non-toxic concentration, at which an alive cell proportion was higher than 95% that was controlled by flow cytometry using PI/annexin staining and trypan blue dye exclusion test. Fluorescent activity of all the PSMs was checked not to have any interference with signals of proteins or antibodies tagged by fluorophores (Supplementary Table 1, Supplementary Fig. 2). All the studied PSMs are DNA-binding compounds, and taking into account the cultural medium volume to calculate the amount of the compound added and the number of cultured cells to calculate the amount of nuclear DNA in the sample, we estimated maximal achievable compound per base ratio (CPBR), however, interactions of PSMs with different proteins were ignored in the calculations. Among 15 PSMs analyzed in our study delphinidin is the only one to demonstrate good solubility in water solutions (Supplementary Fig. 3). The rest 14 PSMs were dissolved in organic solvents (DMSO or ethanol) and added to the reaction mixture or cultural medium of HeLa cells at the concentrations not affecting the studied processes. Final concentrations of the solvents in the cultural medium are also provided in Table 1.

Thus, we demonstrated that all PSMs studied may cause cytotoxic effect and determined maximal non-toxic and IC20 concentrations, which were used in our further study of PSM effects on chromatin structure in cultured HeLa cells.

### 3.2 Nucleosome Stability of HeLa Cells Treated with PSMs

The study of molecular effects caused by PSMs on nucleosome stability was performed using three alternative approaches: (1) analysis of DNA cleavage products of chromatin treatment with micrococcal endonuclease; (2) microscopy of cells with fluorescently labeled H2B histone using HeLa SSRP1-GFP H2B-mCherry cells; (3) measurement of the content changes of the FACT histone chaperone subunits in the nucleoplasm and chromatin-bound protein fractions of HeLa cells using Western blotting.

#### 3.2.1 Nuclear DNA Cleavage by Micrococcal Nuclease (NEB) of Chromatin from the Cells Pretreated with PSMs

**Fig. 1.**
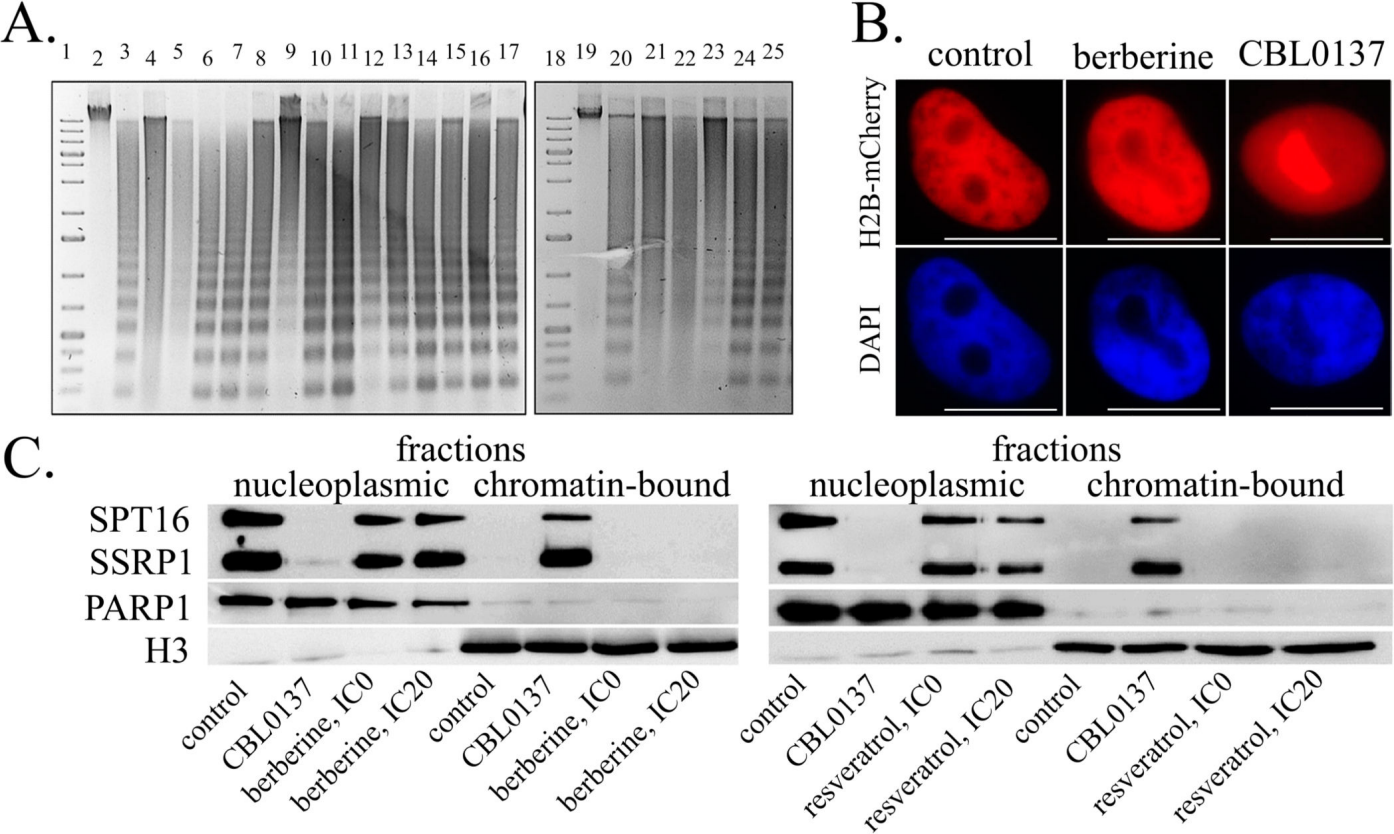
Effects of DNA-binding non-mutagenic plant secondary metabolites (PSMs) on nucleosome stability. (A) Gel electrophoresis of DNA isolated from nuclei of HeLa cells incubated with CBL0137 followed by digestion with micrococcal nuclease. 1, 18—DNA marker; 2, 19—DNA control; 3, 20—negative control; 4, 21—CBL0137, 100 μM; 5, 22—CBL0137, 50 μM; 6—quercetin; 7—coumarin; 8—apigenin; 9—kaempferol; 10—resveratrol; 11—sanguinarine; 12—EGCG; 13—naringenin; 14—genistein; 15—thymoquinone; 16—curcumin; 17—fisetin; 23 ginsenoside Rb1; 24—delphinidin; 25—berberine. (B) HeLa SSRP1-GFP H2B-mCherry cells treated with CBL0137 (50 μM, 1 hour) or berberine (IC0, 24 hours). (C) Western blotting of the nucleoplasmic and chromatin fractions of HeLa cells treated with CBL0137 (50 μM, 1 hour) or PSMs (IC0, 24 hours). Scale bar 10 microns.

Nucleosomal DNA is inaccessible for the micrococcal nuclease cleavage of DNA shielded by nucleosome histones, while the linker regions of DNA are digestible. This leads to DNA fragmentation seen at the electropherograms as the so-called “ordered ladder” (Fig. 1, tracks 3, 20). The lack of this order can be observed only when the cell nuclei are treated with compounds that disrupt the binding of DNA to the nucleosome core histones. CBL0137, which is known to cause chromatin destabilization, was taken as a positive control [35].

After 30 min incubation of HeLa cell nuclei with CBL0137 at concentrations of 50 μM and 100 μM, active DNA cleavage by mnase was observed, while no nucleosome-protected fragments or uncleaved DNA were revealed (Fig. 1A, tracks 4, 5, 21, 22). All the studied PSMs caused the pattern of DNA cleavage showing nucleosome-bound DNA fragments not-digested by NEB (Fig. 1A, tracks 6–17, 23–25).

The choice of concentrations of PSMs used in the reaction mixtures in this experiment was limited by their solubility in water, and we added the compounds even at the cytotoxic concentrations to for HeLa cells. PSM concentrations and CPBRs used in this experiment are provided in Table 2.

#### 3.2.2 Intranuclear Localization of Histone H2B in HeLa Cells Treated with PSMs

Two copies of histone H2B together with two copies each of histones H2A, H3 and H4 form the octamer core of the nucleosome, around which DNA is wrapped. H2B is a canonical histone: it is distributed throughout the chromatin in all phases of the cell cycle. Using *in vivo* microscopy of HeLa cells with fluorescently labeled H2B histone, a complete redistribution of histone H2B from chromatin to the nucleoli was observed after the cells were treated with 50 μM CBL0137 for 1 hour [35]. We used CBL0137 cell treatment as a positive control. Neither berberine nor all the other PSMs at IC20 concentrations after the cells exposition for 1 and 24 hours affected the intranuclear localization of H2B histone, which remained associated with chromatin (Table 2, Fig. 1B).

#### 3.2.3 Intranuclear Localization of Histone Chaperone FACT after HeLa Cell Treatment with PSMs

The FACT histone chaperone is involved in DNA packaging by the complex interaction with core histones when the inner parts of the nucleosome are accessible. This protein is a dimeric subunit of SSRP1 and SPT16. In addition to the interaction of the SPT16 subunit with the H2A/H2B dimer, the SSRP1 FACT subunit interacts with DNA after nucleosome unfolding [35,39]. Using Western-blotting method, we assessed the redistribution of the histone chaperone FACT subunits between the chromatin and nucleoplasmic fractions under the influence of the studied PSMs. CBL0137 was shown to induce FACT trapping on chromatin and we analyzed its influence on SSRP1 and SPT16 redistribution between the chromatin and nucleoplasmic fractions as a positive control. PARP1 of the nucleoplasmic fraction and core histone H3 of the chromatin-associated fraction were taken as reference proteins. At concentrations not exceeding IC20, PSMs did not affect the FACT subunits distribution in the nuclei of HeLa cells both after 1- and 24-hour treatment, while the treatment of the cells with 50 μM CBL0137 for 1 hour caused significant increase in the relative content of SPT16 and SSRP1 subunits in the chromatin fraction and a decrease of their relative content in the nucleoplasm (Fig. 1C).

Thus, using three different approaches we did not reveal PSM influence on nucleosome stability.

### 3.3 Induction of Linker Histones Relocation by PSMs in HeLa Cells

#### 3.3.1 Influence of the PSMs on the Linker Histone H1.5 Localizations in HeLa Cell Nuclei

**Fig. 2.**
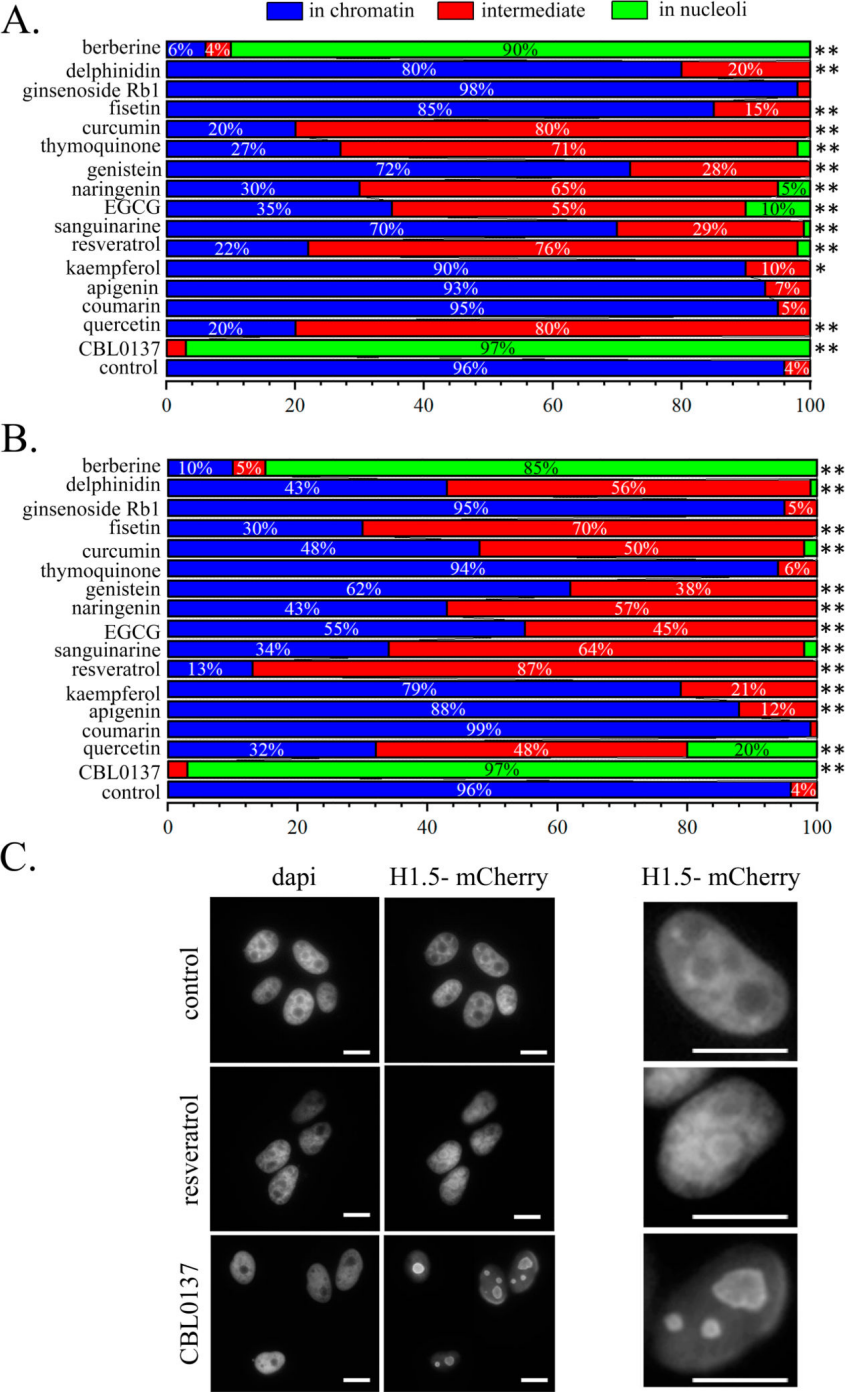
Redistribution of mCherry-tagged linker histone H1.5 under the influence of PSMs. (A) Histogram of the proportions of cells with the different localization of the linker histone H1.5 after 1-hour treatment with CBL0137 or PSMs. (B) Histogram of the proportions of cells with the different localization of the linker histone H1.5 after 1-hour treatment with CBL0137 or 24-hour treatment with PSMs. Statistically significance of the differences between control untreated cells and PSM treated cells by the histone contents in nucleoli and intermediate position (Pearson’s chi-squared test): * — *p* < 0.05, **—*p* < 0.01. (C) Images of the cells after the treatment with 25 μM CBL0137 for 1-hour or resveratrol for 24-hours. Scale bar 10 microns.

We analyzed the localization of the mCherry-tagged linker histone H1.5 in HeLa cells using fluorescent microscopy. Linker histone H1.5 is mainly associated with chromatin and in the cells non-treated with any compounds we observed its distribution over the cell nucleus. When the cells were treated with 25 μM CBL0137 for 1 hour, histone H1.5 was translocated to the nucleoli in almost all the cells (97%). In the cells treated with non-toxic concentrations of PSMs for 1 and 24 hours, similar changes were found when cells were treated with berberine (IC0, 1 hour and 24 hours): in more than 85% of cells, the linker histone H1.5 was translocated to the nucleoli. For the compounds delphinidin, fisetin, genistein, sanguinarine, resveratrol, curcumin, naringenin, quercetin, thymoquinone, and EGCG, a significant change in the proportions of cells with different localization of the linker histone H1.5 (in chromatin, intermediate, in nucleoli) was also demonstrated (Fig. 2).

#### 3.3.2 Depletion of the Linker Histones H1.2 and H1.4 from Chromatin under the Influence of PSMs

**Fig. 3.**
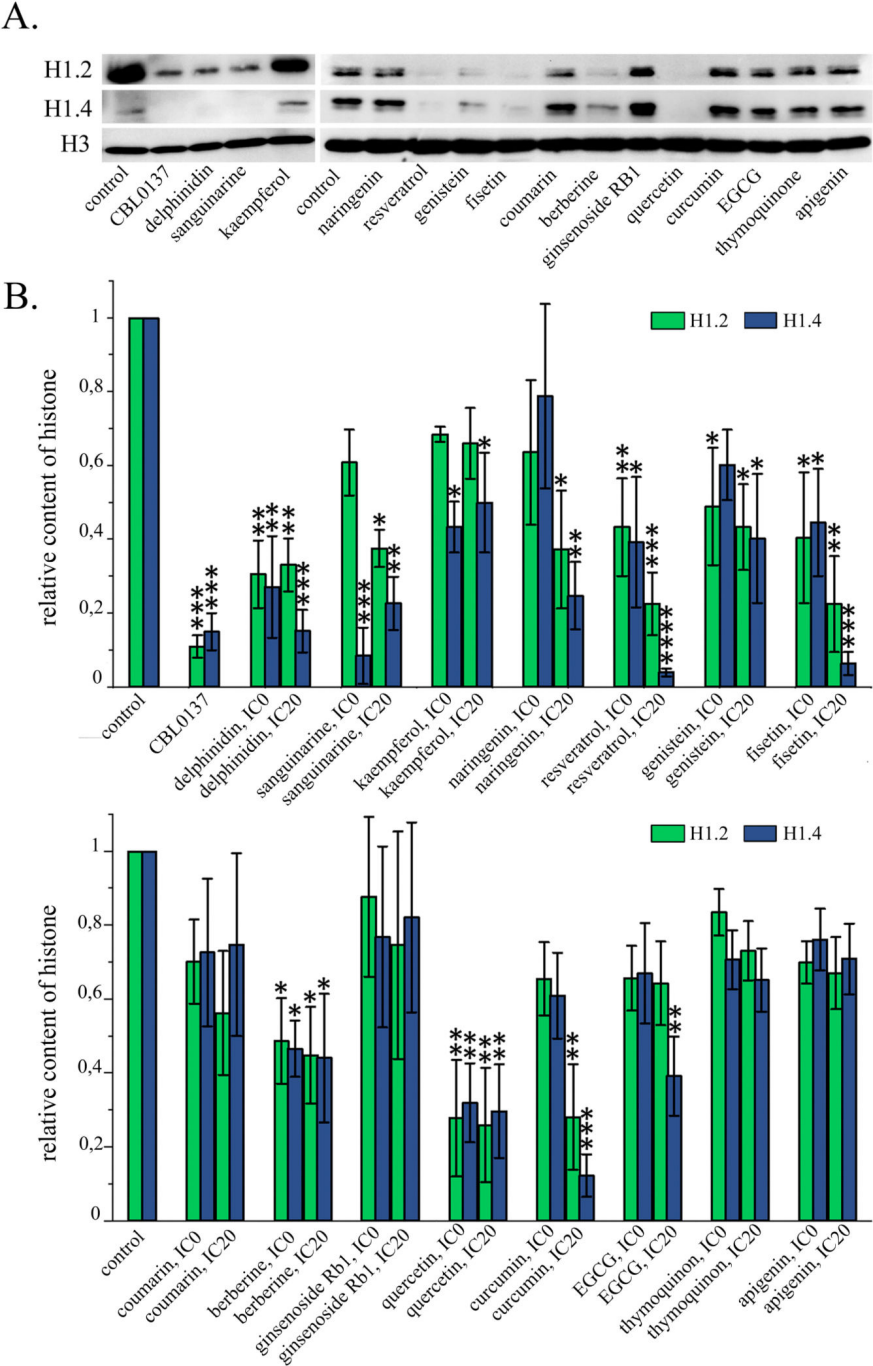
Influence of PSMs on the relative content of H1.2 and H1.4 linker histones in chromatin fraction. (A) Western blotting analysis of the chromatin fraction of HeLa cells treated with PSMs for 24 h. (B) Densitometry analysis of the blots: Mean ± SD; statistically significance of the differences of control untreated cells and PSM treted cells by the relative histone contents (analysis of variance (ANOVA) test and Dunnett’s post hoc test): *—*p* < 0.05, **—*p* < 0.01, ***—*p* < 0.001, ****—*p* < 0.0001.

Having demonstrated a significant change in the nuclear localization of the linker histone H1.5 in the cells exposed to both CBL0137 and a number of PSMs, we took into consideration the following facts: (1) treatment of cells with both CBL0137 and a number of PSMs was demonstrated to induce interferon signaling [34,40], and (2) in the study of Izquierdo-Bouldstridge *et al*. [38], knockdown of the linker histones H1.2 and H1.4 was shown to increase the expression of endogenous retroviruses, accompanied by the triggering of interferon signaling. Accordingly, we suggested that CBL0137 and PSMs can cause depletion from the chromatin fraction of not only histone H1.5, but also other linker histone variants. Since the functional activity of H1.2 and H1.4 histones is associated with the suppression of endogenous retroviruses, on the next stage of our study the effects of CBL0137 and PSMs on the relative content of these linker histone variants in chromatin were analyzed. We also analyzed the effects of PSMs on the relative content of these histone variants in the total pool of cell proteins and on the expression levels of linker histone genes. The relative content of the linker histones H1.2 and H1.4 in the chromatin fraction, as well as in the total pool of cell proteins was assessed using Western blotting followed by densitometry analysis of the blots (Fig. 3, Table 3). Untreated cells served as a negative control.

In the cells treated with 25 μM CBL137 for 1 hour, depletion of histones H1.2 and H1.4 from chromatin fraction was observed, leading to a decrease in their relative content by 9.1 and 6.7 times, respectively. When cells were treated with fisetin, genistein, delphinidin, berberine, sanguinarine, quercetin, and resveratrol at the IC20 and IC0 for 24 hours, we observed significant decrease of both chromatin-bound H1.2 and H1.4 relative contents. The amount of linker histones H1.2 and H1.4 in the chromatin fraction under the influence of the substances at the IC20 decreased, respectively: for fisetin by 4.5 and 16.7 times, genistein—by 2.3 and 2.5 times, delphinidin—by 3.0 and 6.7 times; berberine—by 2.3 and 2.3 times; sanguinarine—2.7 and 4.5 times; quercetin — 4.0 and 3.4 times; resveratrol—4.5 and 33.3 times. With this treatment of the cells with all of the compounds listed above, we did not detect any significant changes in H1.2 and H1.4 relative content in the cell protein pool, with the exception of IC20 resveratrol, which caused a decrease in the relative level of H1.2 in the cell protein pool by 2.3 times. The treatment of cells with resveratrol at the non-toxic concentration led to a statistically significant reduction in the relative content of both H1.2 and H1.4, without any influence on the histones level in the total protein pool of the cells. Curcumin at the IC20, caused a statistically significant decrease in the chromatin bound fraction of the relative content of H1.2 by 3.6 times and H1.4 by 8.3 times, with a slight non-significant decrease of the relative content of these histones in the total protein pool. When cells were exposed to naringenin at the IC20, it also showed a statistically significant decrease in the relative contents of linker histones H1.2 and H1.4 in the chromatin fraction by 2.7 and 4.2 times respectively, however, a decrease of these proteins in the total cell protein pool was also observed: by 1.6 times for H1.2 and by 3.7 times for H1.4. EGCG and kaempferol at the IC20 caused a significant decrease of H1.4 content in the chromatin fraction by 2.6 and 2.0 times respectively, with no changes in the relative content of this histone in the total protein pool. For apigenin, coumarin, ginsenoside, and thymoquinone, there were no statistically significant changes in the relative content of the linker histones H1.2 and H1.4 in the chromatin fraction (Fig. 3).

Possible influence of PSMs on the expression of mRNA of 6 somatic variants of linker histones H1.0, H1.1, H1.2, H1.3, H1.4, and H1.5 was analyzed by RT-qPCR. After 24 hours of cell treatment with PSMs at the IC20 there were no significant changes detected in the relative level of mRNA expression for H1.0, H1.1, H1.2, H1.3, H1.4, and H1.5 (Table 3).

Thus, we reveled significant PSM influence on linker histone localizations. In particular, a 1-hour treatment with 11 PSMs at IC0 induced significant translocation of the linker histone H1.5 to nucleoli; 6 PSMs at IC0 caused statistically significant depletion of the histone H1.2 from chromatin fraction and 3 more PSMs did it at IC20; statistically significant depletion of the histone H1.4 from chromatin fraction was observed for 8 PSM treatment at IC0 and for 3 more PSMs at IC20. Curcumin, resveratrol, berberine, naringenin, and quercetin caused redislocation of all three variants of the studied linker histones showing some overlap of PSM effects on linker histone DNA-binding. To analyse whether PSMs effects on linker histones in chromatin fraction is not specific only for HeLa cells.

We studied influence of resveratrol, genestein, EGCG and CBL0137 on linker histone H1.2 in another cancer cell line—T47D, human breast cancer cells. We observed statistically significant decrease in the amount of this protein in cromatin fraction after 24-hour treatment of the cells with resveratrol at the concentration of 233 μM and genistein at the concentration of 176 μM at 24 hours. In T47D cells 1-hour treatment with CBL0137 at a concentration of 25 μM caused complete depletion of linker histone H1.2 in the chromatin fraction. The data obtained are comparable in two cell lines, that suggest general character of observation independent on cell types (Supplementary Fig. 4).

### 3.4 The Effect of Naringenin, Curcumin and Berberine on the Cell Cycle

Linker histones H1.2 and H1.4 are involved in the regulation of the cell cycle. In particular, linker histone H1.2 depletion induced in T47D cells by shRNA-mediated knockdown was shown to cause cell arrest in the G1 phase and inhibit cell proliferation [41]. In addition, in CBL0137 treatment of the cells of breast cancer and hematological malignancies showed significant changes in cell cycle progression [42,43].

**Fig. 4.**
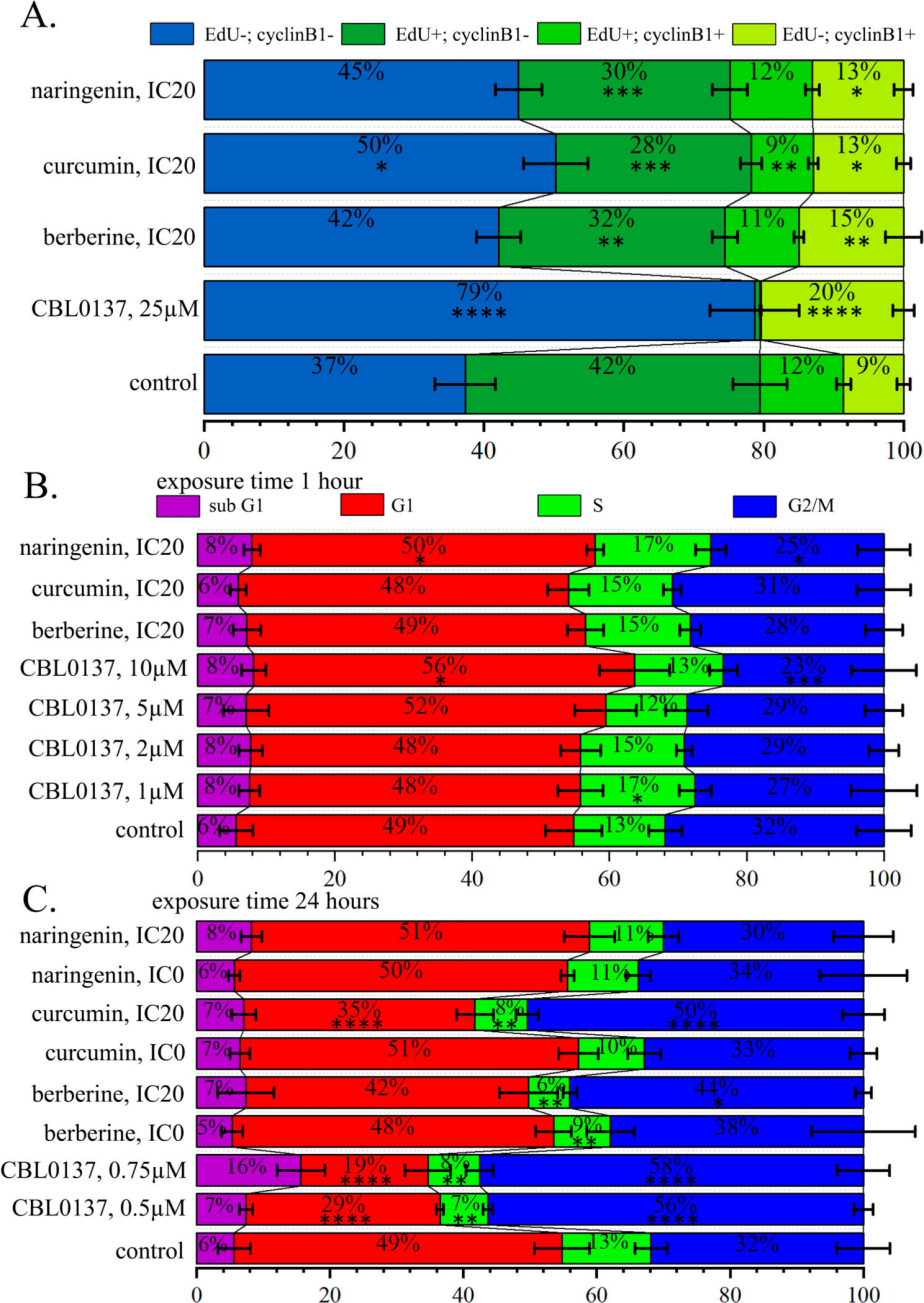
PSM influence on the cell cycle distribution. (A) Histograms of cell proportions detected by 5-Ethynyl-2′-deoxyuridine (EdU) and cyclin B1 markers after 1-hour treatment with CBL0137, berberine, curcumin, and naringenin (M ± SD). (B,C) Histograms of cell cycle distributions based on DNA content (PI staining) after the treatment with CBL0137, berberine, curcumin, and naringenin (M ± SD). (B) After 1-hour treatment. (C) After 24-hour treatment. Significance of the differences between control untreated cells and PSM treated cells was performed using ANOVA test and Dunnett’s post hoc test: *—*p* < 0.05, **— *p* < 0.01, ***—*p* < 0.001, ****—*p* < 0.0001.

PSM influence on cell cycle in HeLa cells was studied for the compounds berberine, naringenin, curcumin, which produced the most significant redistribution of the linker histones H1.5, H1.2 and H1.4. HeLa cells were treated with PSMs at the concentrations IC20 (for 24-hour treatment, Table 1) for 1-hour and then cell populations were analyzed by flow cytometry. Modified thymidine analogue EdU efficiently incorporated into DNA was fluorescently labeled with the bright, photostable dye Alexa Fluor488 in a fast, highly-specific, mild click reaction to detect cells with active DNA synthesis. To mark the G2/M phase, we performed immunofluorescent staining of cells with antibodies to cyclin B1 and then the secondary antibodies AlexaFluor647. Treatment of HeLa cells with CBL0137 caused a significant decrease of the cell proportions (EdU+, cyclin B1–) and (EdU+, cyclin B1+), correspondingly to 0.79% and 0.15% versus 42.10% and 11.95% in the control and increase of the cell proportion (EdU–, cyclin B1–) and (EdU–, cyclin B1+) to 79.20% and 20.53% versus 37.27 ± 4% and 8.57% in the control (Fig. 4A, Supplementary Fig. 5).

Cell treatment with curcumin leads to less intensive but also significant reduction in proportions of the cells incorporating EdU, both cyclin B1– and cyclin B1+, and enlargement of proportions of the cells not incorporating EdU also cyclin B1– and cyclin B1+. Proportions of cells which did not incorporate EdU and both expressing and not expressing cyclin B1 significantly changed ubder influence of all PSMs analysed in this experiment. In particular, after the cell treatment with berberine proportion of cells (EdU–, cyclin B1–) was 42.67%, with curcumin—50.33%, and with naringenin—43.67% against 37.27% in the control cell population. In the untreated cell population, the proportion of cells (EdU–, cyclin B1+) was 8.57%, and it increased after the treatment with with berberine, curcumin, and naringenin up to 15.17%, 12.93% and 12.67%, correspondingly (Fig. 4A, Supplementary Fig. 5).

Significant changes in the proportion of cells incorporting EdU within just 1-hour PSM treatment revealed a decrease in DNA synthesis, and PSM effects were similar to those of CBL0137, although less pronounced.

Analyzing the cell cycle distribution after 1-hour treatment with CBL0137 using PI staining, we observed a statistically significant increase in the proportion of cells in the G1 phase: (55.04%) relative to control (48.73%), and a statistically significant decrease in the proportion of cells in G2/M: phase: (23.18%) compared to control (31.59%). Under naringenin treatment we observed a statistically significant increase in the proportion of cells in S phase (16.75%) relative to control (13.28%) and decrease in G2/M (25.10%) compared to control (31.59%) (Fig. 4B, Supplementary Fig. 6).

Analysis of cell cycle distribution after 24 hours cell treatment with compounds in non-toxic and IC20 concentration using PI staining showed a statistically significant reduction in the proportion of cells in the G1 phase after the treatment with CBL0137 at concentrations of 0.5 μM (28.77%) and 0.75 μM (18.77%) and after the treatment with curcumin at the IC20 concentration (34.18%) relative to control (48.73%). We demonstrated a statistically significant decrease in the proportion of cells in S phase (7.75%) after the treatment with CBL0137 at the concentrations of 0.5 μM (7.00%), 0.75 μM (7.63%) and PSMs: curcumin IC20 (7.74%), berberine IC0 (8.45%), berberine IC20 (6.10%) relative to untreated cells (13.28%) and a statistically significant increase in the proportion of cells in G2/M phase after the treatment with 0.5 μM CBL0137 (54.70%), 0.75 μM CBL0137 (56.81%), berberine IC20 (43.25%) and curcumin IC20 (49.72%) compared to control (31.59%) (Fig. 4C, Supplementary Figs. 5,7).

### 3.5 Influence of Berberine on the Linker Histone H1.5 Nuclear Localizations at Different Cell Cycle Phases

**Fig. 5.**
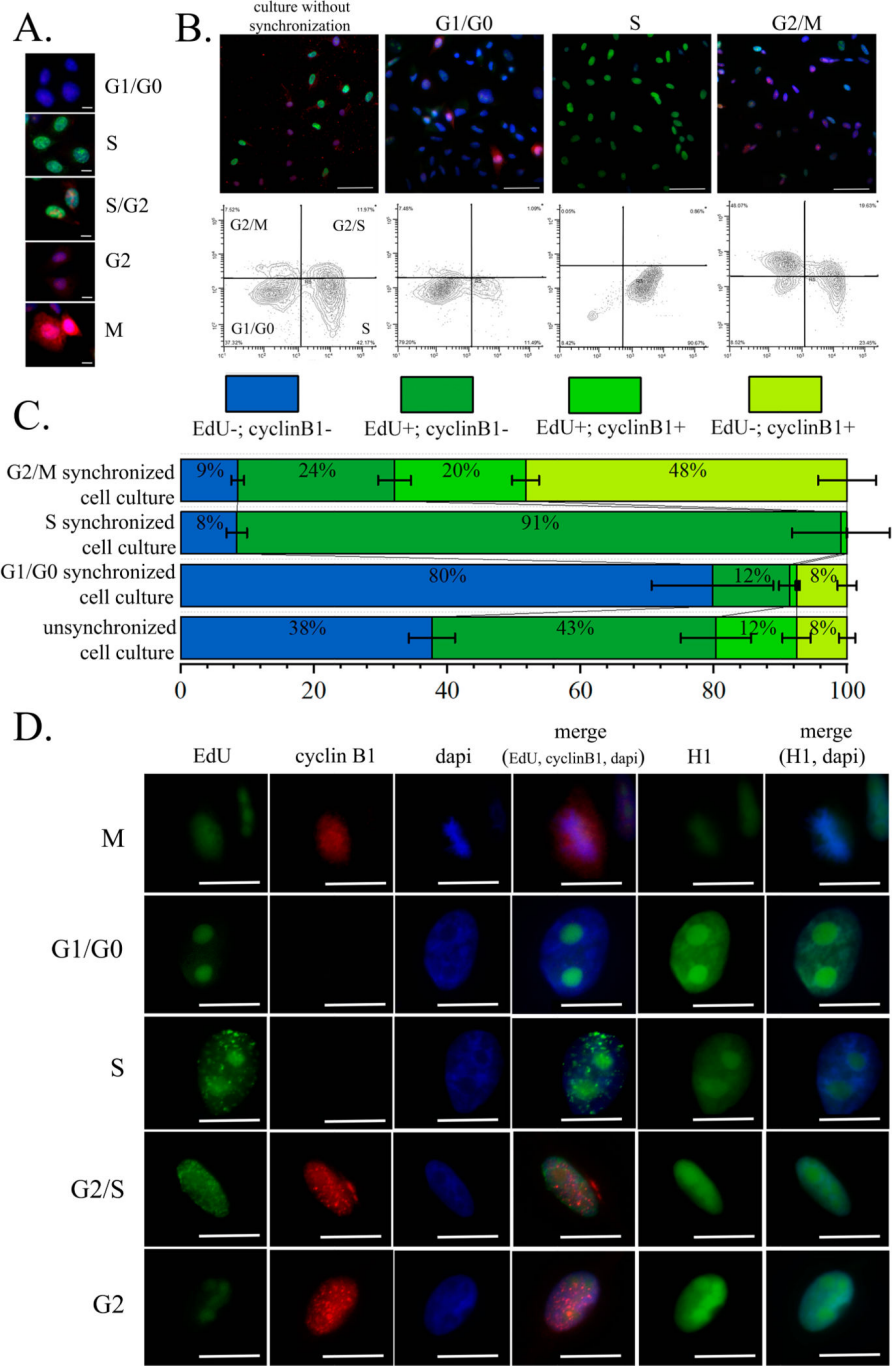
Berberine influence on H1.5 intranuclear translocation in HeLa H1.5-mCherry cells. (A) Fluorescent microscopy of the cells in the different cell cycle phases; Scale bar 10 microns. (B) Results of the fluorescent microscopy and flow cytometry analyses of the cells (1) of the unsynchronized populations, (2) of the population enriched by the cells in G1/G0 phases 14 hours after removal of the thymidine block, (3) of the population enriched by the cells in S phases immediately after removal of the thymidine block, (4) of the population enriched by the cells in G2/M phases 6 hours after removal of the thymidine block; Scale bar 50 microns. (C) The histogram of cell proportions in the unsynchronized population and the populations enriched by the cells in G1/G0-, S-, and G2/M-phases by the thymidine block. (D) Berberine influence on the linker histone H1.5 nuclear localization on the synchronized cell population. Scale bar 10 microns.

Causing linker histone depletion from chromatin, PSMs should influence the degree of DNA packaging. The process of DNA packaging depends on the phase of the cell cycle, and we propose that the effect of PSMs may be different depending on the stage of the cell cycle, at which the treatment occurs. To clarify it, we analyzed the effect of berberine, one of the most active PSM, which induced linker histone H1.5 depletion up to 85% after the only 1-hour treatment, and it also influences proportions of the cells in other cell cycle phases. Immunostaining of the cells and the result interpretation were performed analogous to the analysis at the previous part of the study on the PSM influence on cell cycle progression. Synchronization of the cell cycle of the HeLa H1.5-mCherry cells was performed using a double thymidine block. Since the double thymidine block should lead to cell cycle arrest before S-phase, its removal should be followed by an increase in the number of cells in S-phase compared to the unsynchronized population. Subsequent flow cytometry analysis of the cell population distribution by cell cycle phases after the different periods following the removal of the thymidine block confirmed the enrichment of cell proportions at the different cell cycle phases. Cell cycle distributions were analyzed using EdU-based S-phase detection and cyclin B1-based G2/M-phases detection. The cells without both markers (EdU–, cyclin B1–) were attributed to the G1/G0 phases. According to the data obtained, a significant increase in the proportion of cells in the corresponding phases of the cell cycle was obtained compared to the unsynchronized culture. In particular, after removal of the double thymidine block, the proportion of cells in the S-phase was up to 91 ± 7%, which is more than 2 times higher than that of the unsynchronized culture (42 ± 5%). 6 hours after the removal of the double thymidine block, the cells should pass into the G2/M phase, and indeed, their proportion was up to 48 ± 4%, in contrast to the corresponding cell portion of the unsynchronized culture—7.5 ± 1.2%. And finally, 14 hours after the removal of the double thymidine block, mitosis should already occur and the cells should go into the G1/G0 phase. In the synchronized cell culture, 14 hours after the removal of the thymidine block, the proportion of cells in the G1/G0 phase was up to 79 ± 9%, while in the unsynchronized culture it was 37 ± 3% (Fig. 5A–C).

We have shown that linker histone H1.5 translocation to the nucleoli after the treatment with berberine for occurred at each of the cell cycle phases: G1/G0, S, G2/M, and G2. Thus, no significant changes of berberine effect on the linker histone H1.5 localization depending on the cell cycle phase were found (Fig. 5D).

## 4. Discussion

DNA-binding PSMs are small molecules, which do not form covalent bonds with DNA, but intercalate DNA or interact with DNA grooves and DNA alternative structures (Table 4, Ref. [4,17,19,21,22,25,44–89]).

Recently a different experimental system has shown significant influence of many DNA-binding PSMs with anticancer activity on epigenetic regulation of transcription (Table 4). It is in agreement with an increasing body of data obtained by modern sophisticated technologies revealing that epigenetic enzymes and non-coding part of our genome have key roles in regulation of gene expression, and by this in cell behaviour and carcinogenesis [90,91]. Epigenetic regulation is highly sensitive to environmental signals, which act as “phenotypic-inducer” of defined acquired traits. In particular, a group of small DNA-binding molecules was shown to remodel chromatin structure and activate epigenetically silenced genes [34,44,92]. Moreover, berberine was shown to interact with both DNA and linker histones, which should impact in cooperative interaction with nucleosomes [93]. All the data provided in the Table 4 support our hypothesis that PSMs may remodulate chromatin structure influencing histones interaction with DNA and by this have direct influence on cell transcriptome.

However, analyses of PSM influence on cell chromatin structure have never been performed in spite of the fact of their possible shielding or changing spatial characteristics of the binding sites for histones, epigenetic enzymes, transcription factors, and other DNA-mediated proteins [32,33]. This information is important for profound understanding of the molecular mechanism of PSM anticancer activity and could help to advance cancer chemoprevention drug discovery.

The first goal of our study was to analyze chromatin structure in Hela cells treated with PSMs using approaches revealing both strong nucleosome destabilization and weak effects on DNA-binding of linker histone. We have shown that PSMs, unlike the antitumor drug CBL0137, do not cause any a destabilizing effect on the structure of nucleosomes. None of the studied PSMs affected the pattern of DNA cleavage, when chromatin was treated with micrococcal nuclease. PSMs did not influence localization of the nucleosome core histone H2B tagged by fluorescent mark. Moreover, the relative content of histone chaperone FACT in chromatin fraction measured using Western blotting did not change after HeLa cell treatment with PSMs. Thus, the effects of PSMs on the intranuclear localization of histone H2B and FACT are in good agreement with the data on the absence of the PSM effect on the profile of nuclear DNA cleavage by microccocal nuclease (NEB). By analysis of DNA cleavage products of chromatin treatment with NEB in the positive control, where Hela cell nuclei were treated with CBL0137 for only an hour, we observed entire disappearance of any DNA fragments, which corresponds to previously published data [35]. Moreover, HeLa cell treatment with CBL0137 caused significant redislocation of H2B in nuclei and a significant increase of FACT relative content in chromatin fraction that is in accordance with previously published data concerning CBL0137 effects on chromatin [37,94,95].

We proposed that effects of PSMs on chromatin structure may be significantly weaker compared to CBL0137 as it was demonstrated for indolocarbazole derivative LCS-1269 [44]. Using HeLa cells with mCherry-tagged H1.5 we demonstrated significant redistribution of H1.5 into the nucleoli for 11 PSMs out of 15, in particular, for berberine, delphinidin, fisetin, genistein, sanguinarine, resveratrol, curcumin, naringenin, quercetin, thymoquinone, and EGCG just after 1-hour treatment. The strongest effect was caused by berberine, and this effect is in agreement with the data of Rabbani-Chadegani *et al*. [93], who described cooperative interaction of berberine with DNA, linker histone and nucleosome. Then we analyzed the relative content of H1.2 and H1.4 in chromatin fraction by Western blotting. The reason we chose these linker histone variants to further analyze is they are the predominant variants in most somatic cells additionally, Izquierdo-Bouldstridge *et al*. [38] had shown of these histone variants caused significant transcriptome consequences including activation of endogenous retrovirus expression accompanied by the triggering of interferon signaling. Statistically significant depletion of the histone H1.2 in chromatin fraction of HeLa cells was observed for the treatment with six PSMs at IC0: berberine, delphinidin, fisetin, genistein, quercetin, resveratrol, and three more PSMs, in particular, curcumin, naringenin and sanguinarine, caused H1.2 depletions from chromatin fraction at IC20. Statistically significant depletion of the histone H1.4 in chromatin fraction of HeLa cells was observed for 8 PSM treatment at IC0: berberine, delphinidin, fisetin, genistein, kaempferol, quercetin, resveratrol, and sanguinarine. Three more PSMs, in particular, curcumin, EGCG and naringenin caused H1.4 depletions from chromatin fraction at IC20. The strongest depletion of both linker histone variants from chromatin fraction was observed, when HeLa cells were treated with IC20 fisetin, resveratrol, curcumin, delphinidin and quercetin. The relative protein level of linker histone variants H1.2 and H1.4 in chromatin fraction decreased by 70% or more. Noteworthy, that linker histones H1.2-H1.3-H1.5 depletion by triple knockdown in mouse embryonic stem cells (50% reduction total H1) caused dramatic chromatin structure changes Yuhong Fan *et al*. [96] and depletion of 80–90% of the linker histone variants H1.2 and H1.4 by their knockdown was most deleterious [37]. On the whole, we identified 8 compounds (kaempferol, EGCG, delphinidin, curcumin, genistein and berberine, sanguinarine, fisetin) that cause a statistically significant displacement of linker histones H1.2 and H1.4 from chromatin. Thus, we demonstrated for the first time that some PSMs may realize their effect via depletion of linker histone and that curcumin, resveratrol, EGCG, berberine, naringenin, and quercetin caused depletion of all three variants of linker histones H1.2, H1.4 and H1.5 showing some overlap with PSM effects on linker histone DNA-binding. However, four PSMs, in particular, apigenin, coumarin, ginsenoside Rb1 and thymoquinone, did not cause statistically significant quantitative changes either in the total pool of proteins or in the chromatin-bound protein fraction, which may indicate their low ability to influence the interaction of DNA and linker histones.

We demonstrated that PSMs do not cause any statistically significant effects on the transcription of linker histone genes of six somatic variants. Moreover, the total amount of linker histones H1.2 and H1.4 in cells was not significantly changed, the only two exeptions being resveratrol and naringenin (Table 3). Under the treatment of cells with naringenin and resveratrol, not only a direct depletion of linker histones from chromatin occurs, but other effects on the maturation/degradation of linker proteins in cells also take place. Resveratrol caused depletion of linker histone H1.2 from chromatin fraction up to 4.55 times on the background of decrease in the total amount of linker histones in 2.33 times and significant depletion of linker histones H1.4 (up to 33.33 times) on the background of insignificant decrease of its total amount in nuclei. Naringenin caused significant depletion of linker histones H1.2 and H1.4 from chromatin fraction on the background of insignificant decrease in the total amount of linker histone H1.2 and significant decrease in the total amount of linker histone H1.4. Thus, for most of PSMs depletion of linker histones H1.2 and H1.4 from chromatin fraction was observed on the background of insignificant change in the total pool of H1.2 and H1.4 in nuclei.

Displacement of linker histones from chromatin by PSMs may be proposed to involve either significant competition between PSMs and linker histones for the binding sites of DNA or an influence on the rate of exchange of linker histones with DNA. It can be achieved by increasing post-transcriptional modifications of linker histones, such as phosphorylation, acetylation, formylation, propionylation and crotonylation, leading to a decrease in the positive charge of linker histones that affects DNA packaging and accommodation of a less compact state of chromatin [97,98]. Noteworthy, the PSM effect on H1.5 localization in cell nuclei was observed under 1-hour treatment indicating the most likely competitive inhibition mechanism

Linker histone condensation/decondensation is involved in regulation of cell cycle, DNA repair, thanscription [98–100]. It has been found that relative content of linker histones, their pattern of expression and post-translational modifications in cancer cells are different from characteristics of normal cells and this change is associated with tumor grade and aggressiveness [101]. For example, high expression of H1.5 was found in high-grade tumors (pulmonary neuroendocrine tumors and prostate cancer) against decreased expression of H1.5 observed in low-grade tumors [102,103]. H1.0 is highly expressed in terminally differentiated cells, and its expression is generally downregulated in various cancers [104,105]. Levels of linker histone variants H1.0, H1.1, H1.4 and H1x have been found to be reduced in malignant adenocarcinomas compared to benign adenomas [106,107]. However, on the contrary, high levels of H1.2 expression correlate with poor treatment results for pancreatic cancer [108].

Loss of linker histones or production of a mutant protein can cause aberrant chromatin states. Reduction of H1 stoichiometry leads to decreased H3K27 methylation, increased H3K36 methylation, and intensifies interaction frequency between compartments followed by deregulation of the cell survival pathways [32]. The local density of H1 controls the balance of repressive and active chromatin domains by DNA packaging. Depletion of linker histones may be a marker of genomic instability in tumor cells and be a precursor to more serious transcriptome abnormalities and expression of non-coding regions. A local depletion of some H1 variants was associated with the intensive expression of major satellite and then other components of heterochromatin: LINE, minor satellite, Long terminal repeate (LTR) [109]. In breast cancer cells, coordinated loss of H1.2 and H1.4 was demonstrated to trigger an interferon response due to derepression of heterochromatin regions, that is a characteristic hallmark of many cancers [40]. Noteworthy, that depletion of any linker histone variant induced by the corresponding gene knocked-down is usually compensated by overexpression of other variants, however it does not prevent changes in higher-order chromatin folding [110,111]. Thus, our study data showing that both CBL0137 and a number of DNA-binding PSMs cause the depletion of linker histone variants from chromatin, let us propose their influence on higher-order chromatin folding, enabling LINE, minor satellite, LTR expression and by this activation of interferon I-type signaling.

Linker histones play diverse roles in different cellular processes including cell cycle. Many linker histone variants contain multiple cyclin-dependent kinase (CDK) motifs, which undergo site-specific phosphorylation [97]. These modifications are variant-specific and some of them are highly cell cycle-dependent, reaching a maximum in M phase [112]. Loss of linker histones in chromatin, in addition to transcriptome changing in the expression of a number of genes, can also cause abnormalities in cell cycle progression. Thus, we decided to analyze whether PSMs causing strong depletion of linker histones from chromatin fraction may influence cell cycle distribution. According to our data berberine, curcumin and naringenin influence the cell cycle distribution causing a decrease in DNA synthesis and a significant increase in the proportions of cells in G1/G0 and G2/M phases were observed.

After 1-hour CBL0137 treatment at high concentration (10 μM) we observed significant increase of the proportion of cells in G1-phase that is in agreement with almost entire disappearance of the cells synthesizing DNA (by EdU incorporation): up to 1% against 54% in control untreated cell population. After 1-hour naringenin treatment at the concentration IC20 we observed significant increase of the proportion of cells in S-phase that is also in agreement with significant inhibition of DNA synthesis (by EdU). Significant decrease of the proportion of cells incorporating EdU was also observed under 1-hour cell treatment with curcumin and berberine. We could also conclude that PSMs studied in our cell cycle experiments did not cause fast block before S-phase that opposed to CBL0137 at high concentrations. However, after 24-hour CBL0137 treatment with low concentration we observed significant increase of the proportion of the cells in G2/M-phase; analogous changes also significant but less pronounced, were observed for 24- hour treatments with curcumin and berberine at the concentration of IC20.

These results are in agreement with the data of Sancho *et al*. [41], who showed that in T47D human breast cancer cells knock-down of H1.2 reduces the levels of several proteins required for the cell cycle, including CDK2, MCM2 and PCNA, causing an arrest in the G1 phase of the cell cycle. Moreover, H1.2 depletion inhibits progesterone receptor isoform A (PRA)-mediated cell proliferation and promotes entry into the G2/M and S phases of the cell cycle [113]. It is in accordance with the data on cell arrest in the G0/G1 or G2/M phases shown in a number of studies on cell lines of different cancers, in particular, for berberine in human melanoma A375 and colorectal cancer HCT 116 cells [114,115], curcumin in acute myeloid leukemia K562, colorectal cancer HCT 116, SK-N-SH neuroblastoma and glioblastoma cells [116–119] and naringenin in breast cancer MDA-MB-231 cells [120].

Previously, it was shown that using immunofluorescence labeling by the specific antibodies in synchronized HeLa cells makes phosphorylation of H1.5 Ser17 appear early in G1 at discrete speckles followed by phosphorylation of Ser172. Thr10 phosphorylation started during prophase, showed highest phosphorylation levels during metaphase, and disappeared clearly before chromatin decondensation occurred [112]. Thus, our data demonstrates the translocation of H1.5 into the nucleoli after berberine1-hour treatment of cells in various phases of the cycle for only 1 hour (except mitosis, when chromatin condenses) which may be interpreted as follows: berberine causes concurrent inhibition of linker histone DNA-binding either independently on its post-translational phosphorylation and other post-translational modifications or on those variants of linker histone post-translational modifications that stably present in chromatin.

Functional role of linker histones in the regulation of endogenous retroelements, in particular, LINEs and SINEs, is well known [40,121–123]. In turn, a number of studies have recently revealed involvement of endogenous retroelements in activation of interferon type I signaling, and by this, in both normal physiological functioning of the immune system and pathogenesis of immune disorders [124, 125]. Moreover, inhibition of interferon type I signaling was shown to immunosuppress tumor microenvironment while its restoring is followed by antitumor immunity activation [126]. Herein, we demonstrated for the first time that PSMs cause linker histone depletion from chromatin fraction, that may represent a possible mechanism for induction of interferon type I signaling, which has been described for a number of PSMs, and it represents one of the possible mechanisms of PSM anticancer activity.

## 5. Conclusions

Recently, great progress has been achieved in understanding of chromatin organization and functioning. In particular, the role of linker histones, the most abundant chromatin-associated proteins, was demonsrated in the regulation of such dynamic processes in cells as replication, cell cycle, DNA repair, RNA turnover, transcription and development was demonstrated. Affinity of every linker histone variant for chromatin depends on its post-translational modifications occurring in various positions and differing by several types, that finally determines accessibility of the DNA region for other DNA-mediated proteins. It explains diverse and complex peculiarities of linker histone functions both in the open and closed states of chromatin. In our study we demonstrated PSM influence on interaction of DNA with linker histones H1.2, H1.4 and H1.5 that is significant for understanding of xenobiotic influence on genome functioning and for elucidation of the mechanism of anticancer effect of PSM. In future it will be important to find out influence of PSM on DNA interaction with other variants of linker histones and studied variants possessing different post-translational modifications. Linker histone variants H1.2 and H1.4 involvement in regulations of expression of LINEs and SINEs, influencing interferon type I signaling, demonstrates how linker histones may impact physiological functioning of the immune system. In this context our finding of the ability of PSMs to cause H1.2, H1.4 and H1.5 translocations from chromatin not only demonstrates one of the possible mechanisms of PSM biological activity, but also reveal the way how small DNA binding molecules may cause transcriptome adaptive changes. That extends our understanding of the complex peculiarities of linker histone functioning and opens a new approach for their further studies, in particular, their influence on LINE expression in respect to immune control of cancer cell clone formation.

## Supplementary Material

Supplementary Materials

Supplementary Material

Supplementary material associated with this article can be found, in the online version, at 10.31083/j.fbl2908275.

## Figures and Tables

**Table 1.** Cytotoxicity of PSMs against HeLa cells after 24-hour exposure.

№	Compound	IC50 (μM)	IC20 (μM)	Solvent (%)	CPBR	IC0 (μM)	Solvent (%)	CPBR
1	apigenin	52 ± 9	12 ± 2	0.01 DMSO	0.5	5	≤0.01% DMSO	≤0.2
2	berberine	709 ± 48	108 ± 11	0.15% DMSO	4.3	10	≤0.03% DMSO	≤0.4
3	coumarin	1868 ± 98	860 ± 66	0.21% EtOH	34.4	260	≤0.06% EtOH	≤10.4
4	curcumin	32 ± 2	15 ± 1	0.02% DMSO	0.6	7.5	≤0.01% DMSO	≤0.3
5	delphinidin	874 ± 78	284 ± 34	water	11.4	100	water	≤4
6	EGCG	282 ± 28	133 ± 20	0.14% EtOH	5.3	65	≤0.07% EtOH	≤2.6
7	fisetin	553 ± 45	118 ± 13	0.06% DMSO	4.7	27	≤0.01% DMSO	≤1.1
8	genistein	842 ± 43	176 ± 18	0.18% DMSO	7	60	≤0.06% DMSO	≤2.4
9	ginsenoside Rb1	285 ± 22	80 ± 4	0.09% DMSO	3.2	30	≤0.03% DMSO	≤1.2
10	kaempferol	115 ± 9	10 ± 2	0.01% DMSO	0.4	2	≤0.01% DMSO	≤0.08
11	naringenin	457 ± 17	236 ± 11	0.19% EtOH	9.4	52	≤0.04% DMSO	≤2.1
12	quercetin	109 ± 6	34 ± 3	0.06% EtOH	1.4	10	≤0.02% EtOH	≤0.4
13	resveratrol	707 ± 57	233 ± 24	0.11% EtOH	9.3	50	≤0.02% EtOH	≤2
14	sanguinarine	4.0 ± 0.2	2.1 ± 0.2	0.02% DMSO	0.08	0.8	≤0.01% DMSO	≤0.03
15	thymoquinone	33 ± 1	18 ± 1	0.02% DMSO	0.7	3	≤0.01% DMSO	≤0.12

PSMs, plant secondary metabolites; EGCG, epigallocatechin gallate; CPBR, compound per base ratio; DMSO, dimethyl sulfoxide; EtOH, ethanol.

**Table 2.** Concentrations of DNA-binding compounds used in the experiment with micrococcal nuclease.

№	Compound	Concentration (μM)	CPBR	Solvent*
1	CBL0137	100	1.5	DMSO
2	CBL0137	50	0.8	DMSO
3	apigenin	460	6.7	DMSO
4	berberine	370	5.4	DMSO
5	coumarin	2000	30.0	EtOH
6	curcumin	340	4.9	DMSO
7	delphinidin	300	4.3	DMSO
8	EGCG	450	6.6	EtOH
9	fisetin	1000	14.5	DMSO
10	genistein	500	7.3	DMSO
11	ginsenoside Rb1	450	6.7	DMSO
12	kaempferol	440	6.3	DMSO
13	naringenin	610	8.9	EtOH
14	quercetin	275	4.0	EtOH
15	resveratrol	1050	15.2	EtOH
16	sanguinarine	50	0.8	DMSO
17	thymoquinone	380	5.5	EtOH

* solvents were used in concentrations 0.4% or less (up to 0%).

**Table 3.** Influence of the PSMs on the relative content of H1.2 and H1.4 linker histones in chromatin fraction, cell protein pool and on the relative level of H1.2 and H1.4 gene expression*.

#	Compound	Concentration	Chromatin fraction		Total cell protein pool		mRNA level of the linker histones					

			H1.2	H1.4	H1.2	H1.4	1	1.1	1.2	1.3	1.4	1.5
1	-(control)	-	1	1	1	1	1	1	1	1	1	1
2	CBL0137	25 μM	0.11 ± 0.03	0.15 ± 0.05	0.75 ± 0.04	0.77 ± 0.12						
3	apigenin	IC0	0.69 ±0.05	0.76 ± 0.08								
		IC20	0.67 ± 0.09	0.71 ± 0.09	1.19 ± 0.07	0.81 ± 0.17	1.17 ± 0.21	0.92 ± 0.09	0.77 ± 0.16	1.45 ± 0.42	1.03 ± 0.05	0.92 ± 0.11
4	berberine	IC0	0.45 ± 0.11	0.46 ± 0.13								
		IC20	0.44 ± 0.13	0.44 ± 0.17	0.97 ± 0.13	0.57 ± 0.18	1.73 ± 0.46	0.91 ± 0.18	1.26 ± 0.44	1.15 ± 0.25	1.46 ± 0.24	1.24 ± 0.22
5	coumarin	IC0	0.70 ± 0.11	0.72 ± 0.19								
		IC20	0.56 ± 0.16	0.74 ± 0.24	1.18 ± 0.16	0.86 ± 0.21	0.93 ± 0.27	1.44 ± 0.43	1.14 ± 0.34	1.36 ± 0.14	1.30 ± 0.07	1.38 ± 0.24
6	curcumin	IC0	0.65 ± 0.09	0.60 ± 0.11								
		IC20	0.28 ± 0.14	0.12 ± 0.05	0.97 ± 0.05	0.73 ± 0.20	1.13 ± 0.33	1.26 ± 0.28	0.9 ± 0.08	1.19 ± 0.2	1.36 ± 0.09	1.11 ± 0.2
7	delphinidin	IC0	0.30 ± 0.09	0.27 ± 0.13								
		IC20	0.33 ± 0.07	0.15 ± 0.05	0.89 ± 0.14	0.89 ± 0.10	1.06 ± 0.1	0.82 ± 0.10	1.39 ± 0.21	0.73 ± 0.29	0.82 ± 0.13	0.80 ± 0.10
8	EGCG	IC0	0.65 ± 0.08	0.67 ± 0.13								
		IC20	0.64 ± 0.11	0.39 = 0.1	1.01 ± 0.2	1.00 ± 0.13	1.75 ± 0.32	1.56 ± 0.41	1.30 ± 0.21	1.12 ± 0.37	1.35 ± 0.36	1.34 ± 0.19
9	fisetin	IC0	0.40 ± 0.17	0.44 ± 0.14								
		IC20	0.22 ± 0.13	0.06 ± 0.03	1.31 ± 0.08	0.58 ± 0.16	1.58 ± 0.51	0.73 ± 0.14	0.87 ± 0.25	0.74 ± 0.22	1.6 ± 0.40	0.92 ±0.17
10	genistein	IC0	0.48 ± 0.15	0.60 ± 0.09								
		IC20	0.43 ± 0.11	0.40 ± 0.12	0.78 ± 0.09	0.57 ± 0.15	1.68 ± 0.24	0.85 ± 0.2	1.21 ± 0.25	0.68 ± 0.33	0.74 ± 0.23	0.90 ± 0.17
11	ginsenoside Rb1	IC0	0.87 ± 0.21	0.76 ± 0.24								
		IC20	0.74 ± 0.3	0.82 ± 0.25	0.87 ± 0.28	0.79 ± 0.11	1.43 ± 0.42	0.82 ± 0.18	0.83 ± 0.12	1.12 ± 0.02	1.14 ± 0.09	1.55 ± 0.14
12	kaempferol	IC0	0.68 ± 0.02	0.43 ± 0.06								
		IC20	0.66 ± 0.09	0.49 ± 0.13	1.13 ± 0.17	1.02 ± 0.31	1.06 ± 0.36	0.95 ± 0.09	1.14 ± 0.31	0.94 ± 0.08	1.47 ± 0.21	1.25 ± 0.13
13	naringenin	IC0	0.63 ± 0.19	0.78 ± 0.24								
		IC20	0.37 ± 0.15	0.24 ± 0.09	0.60 ± 0.11	0.27 ± 0.05	1.13 ± 0.18	1.47 ± 0.68	1.21 ± 0.14	1.08 ± 0.18	1.29 ± 0.23	1.34 ± 0.06
14	quercetin	IC0	0.27 ± 0.15	0.32 ± 0.10								
		IC20	0.25 ± 0.15	0.29 ± 0.12	1.57 ± 0.37	0.84 ± 0.23	1.40 ± 0.22	1.13 ± 0.16	0.97 ± 0.21	1.19 ± 0.1	1.54 ± 0.29	1.20 ±0.13
15	resveratrol	IC0	0.43 ± 0.13	0.39 ± 0.17								
		IC20	0.22 ± 0.08	0.03 ± 0.01	0.43 ± 0.06	1 0.60 ± 0.21	0.99 ± 0.16	1.30 ± 0.40	1.37 ± 0.25	0.96 ± 0.35	0.91 ± 0.47	0.63 ± 0.06
16	sanguinarine	IC0	0.60 ± 0.08	0.08 ± 0.07								
		IC20	0.37 ± 0.05	0.22 ± 0.07	1.49 ± 0.25	0.59 ± 0.13	1.01 ± 0.67	1.40 ± 0.21	1.33 ± 0.39	1.13 ± 0.23	1.02 ± 0.11	1.06 ± 0.18
17	thymoquinone	IC0	0.83 ± 0.06	0.70 ± 0.07								
		IC20	0.73 ± 0.08	0.65 ± 0.08	0.83 ± 0.22	0.86 ± 0.11	0.99 ± 0.04	1.01 ± 0.27	0.77 ± 0.13	1.77 ± 0.54	1.62 ± 0.46	1.33 ± 0.17

* The differences were estimated for control untreated cells and PSM treated cells. Red: statistically significant decrease, rose: less than 0.6 decrease; Dark blue: statistically significant increase, blue: more than 1.4 increase.

**Table 4.** Plant secondary metabolite DNA-binding and epigenetic activity.

№	Compound	Effect	Reference
		DNA-binding (intercalation)	[19,45]
1	Apigenin	DNA-binding (minor groove)	[46]
		Epigenetic activation (inhibition HDAC)	[45,47,48]
		Epigenetic activation (inhibition DNMT)	[45]

		DNA-binding (intercalation)	[49]
2	Berberine	DNA-binding (minor groove)	[50]
		Epigenetic activation (inhibition HDAC)	[51]
		Epigenetic activation (inhibition DNMT)	[52]

3	Coumarin	DNA-binding (minor groove)	[53,54]

		DNA-binding (intercalation)	[46]
4	Curcumin	DNA-binding (minor groove)	[17,44,55,56]
		Epigenetic enzyme 1 (inhibitor HDAC)	[48,56]
		Epigenetic enzyme 2 (inhibitor DNMT)	[48,57,58]

		DNA-binding (intercalation)	[74]
5	Delphinidin	DNA-binding (minor groove)	[75]
		Epigenetic activation (inhibition HDAC)	[59,60]
		Epigenetic activation (inhibition DNMT)	[61]

		DNA-binding (intercalation)	[21,25,62,63]
6	EGCG	DNA-binding (minor groove)	[21]
		Epigenetic activation (inhibition HDAC)	[58,64–67]
		Epigenetic activation (inhibition DNMT)	[57,58,65,66]

		DNA-binding (intercalation)	[4,68]
7	Fisetin	Epigenetic activation (inhibition HDAC)	[69]
		Epigenetic activation (inhibition DNMT)	[70]

		DNA-binding (intercalation)	[47]
8	Genistein	DNA-binding (minor groove)	
		Epigenetic activation (inhibition HDAC)	[48,71]
		Epigenetic activation (inhibition DNMT)	[71]

9	Ginsinoside	DNA-binding (minor groove)	[72]
	Rb1	Epigenetic activation (inhibition HDAC)	[73]

		DNA-binding (intercalation)	[74]
10	Kaempferol	DNA-binding (minor groove)	
		Epigenetic activation (inhibition HDAC)	[75]
		Epigenetic activation (inhibition DNMT)	[76,77]

		DNA-binding (intercalation)	[4,19]
11	Naringenin	DNA-binding (minor groove)	[46]
		Epigenetic activation (inhibition HDAC)	[78,79]
		Epigenetic activation (inhibition DNMT)	[80]

		DNA-binding (intercalation)	[74]
12	Qeurcetin	DNA-binding (minor groove)	[81]
		Epigenetic activation (inhibition HDAC)	[48,58,82]
		Epigenetic activation (inhibition DNMT)	[55,58,82]

		DNA-binding (intercalation)	[17,81,83]
13	Resveratrol	DNA-binding (minor groove)	[17]
		Epigenetic activation (inhibition HDAC)	[48,58,84]
		Epigenetic activation (inhibition DNMT)	[57,58,84]

		DNA-binding (intercalation)	[22]
14	Sanguinarine	DNA-binding (minor groove)	[85]
		Epigenetic activation (inhibition HDAC)	[86]
		Epigenetic activation (inhibition DNMT)	

		DNA-binding (intercalation)	[60]
15	Thymoquinone	DNA-binding (minor groove)	[87]
		Epigenetic activation (inhibition HDAC)	[88,89]
		Epigenetic activation (inhibition DNMT)	[88]

HDAC, Histone deacetylase; DNMT, DNA methyltransferase.

## Data Availability

Data presented in this study are contained within this article and in the supplementary materials, or are available upon request to the corresponding author.

## Abbreviations

PSMs: plant secondary metabolites
MGBL: minor groove binding ligands
CDK: cyclin-dependent kinase
DMEM: Dulbecco’s Modified Eagle Medium

## References

[1] Mentella MC, Scaldaferri F, Ricci C, Gasbarrini A, Miggiano GAD. Cancer and Mediterranean Diet: A Review. Nutrients. 2019; 11: 2059. 31480794 10.3390/nu11092059 PMC6770822

[2] Fantini M, Benvenuto M, Masuelli L, Frajese GV, Tresoldi I, Modesti A, *In vitro* and *in vivo* antitumoral effects of combinations of polyphenols, or polyphenols and anticancer drugs: perspectives on cancer treatment. International Journal of Molecular Sciences. 2015; 16: 9236–9282. 25918934 10.3390/ijms16059236 PMC4463587

[3] Jantan I, Ahmad W, Bukhari SNA. Plant-derived immunomodulators: an insight on their preclinical evaluation and clinical trials. Frontiers in Plant Science. 2015; 6: 655. 26379683 10.3389/fpls.2015.00655 PMC4548092

[4] Pezzuto JM. Resveratrol: Twenty Years of Growth, Development and Controversy. Biomolecules & Therapeutics. 2019; 27: 1–14. 30332889 10.4062/biomolther.2018.176 PMC6319551

[5] Russo M, Russo GL, Daglia M, Kasi PD, Ravi S, Nabavi SF, Understanding genistein in cancer: The “good” and the “bad” effects: A review. Food Chemistry. 2016; 196: 589–600. 26593532 10.1016/j.foodchem.2015.09.085

[6] Barnes S Effect of genistein on *in vitro* and *in vivo* models of cancer. The Journal of Nutrition. 1995; 125: 777S–783S. 7884564 10.1093/jn/125.3_Suppl.777S

[7] Bishayee A Cancer prevention and treatment with resveratrol: from rodent studies to clinical trials. Cancer Prevention Research. 2009; 2: 409–418. 19401532 10.1158/1940-6207.CAPR-08-0160

[8] Kisková T, Ekmekcioglu C, Garajová M, Orendáš P, Bojková B, Bobrov N, A combination of resveratrol and melatonin exerts chemopreventive effects in N-methyl-N-nitrosourea-induced rat mammary carcinogenesis. European Journal of Cancer Prevention. 2012; 21: 163–170. 22044852 10.1097/CEJ.0b013e32834c9c0f

[9] Whitsett TG Jr, Lamartiniere CA. Genistein and resveratrol: mammary cancer chemoprevention and mechanisms of action in the rat. Expert Review of Anticancer Therapy. 2006; 6: 1699–1706. 17181483 10.1586/14737140.6.12.1699

[10] Chen YX, Gao QY, Zou TH, Wang BM, Liu SD, Sheng JQ, Berberine versus placebo for the prevention of recurrence of colorectal adenoma: a multicentre, double-blinded, randomised controlled study. The Lancet. Gastroenterology & Hepatology. 2020; 5: 267–275. 31926918 10.1016/S2468-1253(19)30409-1

[11] Zhang H, Gordon R, Li W, Yang X, Pattanayak A, Fowler G, Genistein treatment duration effects biomarkers of cell motility in human prostate. PLoS ONE. 2019; 14: e0214078. 10.1371/journal.pone.0214078 PMC6436751 30917169

[12] Thomas R, Williams M, Sharma H, Chaudry A, Bellamy P. A double-blind, placebo-controlled randomised trial evaluating the effect of a polyphenol-rich whole food supplement on PSA progression in men with prostate cancer–the U.K. NCRN Pomi-T study. Prostate Cancer and Prostatic Diseases. 2014; 17: 180–186. 24614693 10.1038/pcan.2014.6 PMC4020278

[13] Britton RG, Kovoor C, Brown K. Direct molecular targets of resveratrol: identifying key interactions to unlock complex mechanisms. Annals of the New York Academy of Sciences. 2015; 1348: 124–133. 26099829 10.1111/nyas.12796

[14] Khan F, Niaz K, Maqbool F, Ismail Hassan F, Abdollahi M, Nagulapalli Venkata KC, Molecular Targets Underlying the Anticancer Effects of Quercetin: An Update. Nutrients. 2016; 8: 529. 27589790 10.3390/nu8090529 PMC5037516

[15] Nagaraju GP, Zafar SF, El-Rayes BF. Pleiotropic effects of genistein in metabolic, inflammatory, and malignant diseases. Nutrition Reviews. 2013; 71: 562–572. 23865800 10.1111/nure.12044

[16] Qadir MI, Naqvi STQ, Muhammad SA. Curcumin: a Polyphenol with Molecular Targets for Cancer Control. Asian Pacific Journal of Cancer Prevention. 2016; 17: 2735–2739. 27356682

[17] N’soukpoé-Kossi CN, Bourassa P, Mandeville JS, Bekale L, Tajmir-Riahi HA. Structural modeling for DNA binding to antioxidants resveratrol, genistein and curcumin. Journal of Photochemistry and Photobiology. B, Biology. 2015; 151: 69–75. 26188387 10.1016/j.jphotobiol.2015.07.007

[18] Kanakis CD, Tarantilis PA, Polissiou MG, Tajmir-Riahi H-A. Interaction of Antioxidant Flavonoids with tRNA: Intercalation or External Binding and Comparison with Flavonoid-DNA Adducts. DNA and Cell Biology. 2006; 25: 116–123. 16460235 10.1089/dna.2006.25.116

[19] Nafisi S, Hashemi M, Rajabi M, Tajmir-Riahi HA. DNA adducts with antioxidant flavonoids: morin, apigenin, and naringin. DNA and Cell Biology. 2008; 27: 433–442. 18491957 10.1089/dna.2008.0735

[20] Bhattacharjee S, Chakraborty S, Sengupta PK, Bhowmik S. Exploring the Interactions of the Dietary Plant Flavonoids Fisetin and Naringenin with G-Quadruplex and Duplex DNA, Showing Contrasting Binding Behavior: Spectroscopic and Molecular Modeling Approaches. The Journal of Physical Chemistry. B. 2016; 120: 8942–8952. 27491376 10.1021/acs.jpcb.6b06357

[21] Galindo-Murillo R, Cheatham TE 3rd. Computational DNA binding studies of (−)-epigallocatechin-3-gallate. Journal of Biomolecular Structure & Dynamics. 2018; 36: 3311–3323. 29059014 10.1080/07391102.2017.1389306

[22] Khurana S, Kukreti S, Kaushik M. Designing a two-stage colorimetric sensing strategy based on citrate reduced gold nanoparticles: Sequential detection of Sanguinarine (anticancer drug) and visual sensing of DNA. Spectrochimica Acta. Part A, Molecular and Biomolecular Spectroscopy. 2021; 246: 119039. 10.1016/j.saa.2020.119039 33080515

[23] Basu A, Kumar GS. Biophysical studies on curcumindeoxyribonucleic acid interaction: spectroscopic and calorimetric approach. International Journal of Biological Macromolecules. 2013; 62: 257–264. 24041996 10.1016/j.ijbiomac.2013.09.003

[24] Pandya N, Khan E, Jain N, Satham L, Singh R, Makde RD, Curcumin analogs exhibit anti-cancer activity by selectively targeting G-quadruplex forming c-myc promoter sequence. Biochimie. 2021; 180: 205–221. 33188859 10.1016/j.biochi.2020.11.006

[25] Mikutis G, Karaköse H, Jaiswal R, LeGresley A, Islam T, Fernandez-Lahore M, Phenolic promiscuity in the cell nucleus–epigallocatechingallate (EGCG) and theaflavin-3,3’-digallate from green and black tea bind to model cell nuclear structures including histone proteins, double stranded DNA and telomeric quadruplex DNA. Food & Function. 2013; 4: 328–337. 23172122 10.1039/c2fo30159h

[26] Bhattacharjee S, Chakraborty S, Chorell E, Sengupta PK, Bhowmik S. Importance of the hydroxyl substituents in the B-ring of plant flavonols on their preferential binding interactions with VEGF G-quadruplex DNA: Multi-spectroscopic and molecular modeling studies. International Journal of Biological Macromolecules. 2018; 118: 629–639. 29953891 10.1016/j.ijbiomac.2018.06.115

[27] Dickerhoff J, Brundridge N, McLuckey SA, Yang D. Berberine Molecular Recognition of the Parallel MYC G-Quadruplex in Solution. Journal of Medicinal Chemistry. 2021; 64: 16205–16212. 34677968 10.1021/acs.jmedchem.1c01508 PMC8614230

[28] Jarosova P, Paroulek P, Rajecky M, Rajecka V, Taborska E, Eritja R, Naturally occurring quaternary benzo[c]phenanthridine alkaloids selectively stabilize G-quadruplexes. Physical Chemistry Chemical Physics: PCCP. 2018; 20: 21772–21782. 30106067 10.1039/c8cp02681e

[29] Tawani A, Mishra SK, Kumar A. Structural insight for the recognition of G-quadruplex structure at human c-myc promoter sequence by flavonoid Quercetin. Scientific Reports. 2017; 7: 3600. 28620169 10.1038/s41598-017-03906-3 PMC5472631

[30] Salem AA, El Haty IA, Abdou IM, Mu Y. Interaction of human telomeric G-quadruplex DNA with thymoquinone: a possible mechanism for thymoquinone anticancer effect. Biochimica et Biophysica Acta. 2015; 1850: 329–342. 25450185 10.1016/j.bbagen.2014.10.018

[31] Zenkov RG, Kirsanov KI, Ogloblina AM, Vlasova OA, Naberezhnov DS, Karpechenko NY, Effects of G-Quadruplex-Binding Plant Secondary Metabolites on *c-MYC* Expression. International Journal of Molecular Sciences. 2022; 23: 9209. 36012470 10.3390/ijms23169209 PMC9409388

[32] Willcockson MA, Healton SE, Weiss CN, Bartholdy BA, Botbol Y, Mishra LN, H1 histones control the epigenetic landscape by local chromatin compaction. Nature. 2021; 589: 293–298. 33299182 10.1038/s41586-020-3032-z PMC8110206

[33] Yang Z, Sun J, Hu Y, Wang F, Wang X, Qiao HH, Histone H1 defect in escort cells triggers germline tumor in Drosophila ovary. Developmental Biology. 2017; 424: 40–49. 28232075 10.1016/j.ydbio.2017.02.012

[34] Leonova K, Safina A, Nesher E, Sandlesh P, Pratt R, Burkhart C, TRAIN (Transcription of Repeats Activates INterferon) in response to chromatin destabilization induced by small molecules in mammalian cells. eLife. 2018; 7: e30842. 10.7554/eLife.30842 PMC5815852 29400649

[35] Safina A, Cheney P, Pal M, Brodsky L, Ivanov A, Kirsanov K, FACT is a sensor of DNA torsional stress in eukaryotic cells. Nucleic Acids Research. 2017; 45: 1925–1945. 28082391 10.1093/nar/gkw1366 PMC5389579

[36] Kirsanov KI, Kotova E, Makhov P, Golovine K, Lesovaya EA, Kolenko VM, Minor grove binding ligands disrupt PARP-1 activation pathways. Oncotarget. 2014; 5: 428–437. 24504413 10.18632/oncotarget.1742 PMC3964218

[37] Chang HW, Valieva ME, Safina A, Chereji RV, Wang J, Kulaeva OI, Mechanism of FACT removal from transcribed genes by anticancer drugs curaxins. Science Advances. 2018; 4: eaav2131. 10.1126/sciadv.aav2131 PMC6221510 30417101

[38] Izquierdo-Bouldstridge A, Bustillos A, Bonet-Costa C, Aribau-Miralbés P, García-Gomis D, Dabad M, Histone H1 depletion triggers an interferon response in cancer cells via activation of heterochromatic repeats. Nucleic Acids Research. 2017; 45: 11622–11642. 28977426 10.1093/nar/gkx746 PMC5714221

[39] Tsunaka Y, Fujiwara Y, Oyama T, Hirose S, Morikawa K. Integrated molecular mechanism directing nucleosome reorganization by human FACT. Genes & Development. 2016; 30: 673–686. 10.1101/gad.274183.115 PMC4803053 26966247

[40] Vlasova OA, Borunova AA, Safina A, Smetanina IV, Lesovaya EA, Belitsky GA, Activation of interferon-α signaling by resveratrol, genistein and quercetin. Siberian Journal of Oncology. 2019; 18: 50–55.

[41] Sancho M, Diani E, Beato M, Jordan A. Depletion of human histone H1 variants uncovers specific roles in gene expression and cell growth. PLoS Genetics. 2008; 4: e1000227. 10.1371/journal.pgen.1000227 PMC2563032 18927631

[42] Fetisov TI, Borunova AA, Antipova AS, Antoshina EE, Trukhanova LS, Gorkova TG, Targeting Features of Curaxin CBL0137 on Hematological Malignancies In Vitro and In Vivo. Biomedicines. 2023; 11: 230. 36672738 10.3390/biomedicines11010230 PMC9856019

[43] Fleyshman D, Prendergast L, Safina A, Paszkiewicz G, Commane M, Morgan K, Level of FACT defines the transcriptional landscape and aggressive phenotype of breast cancer cells. Oncotarget. 2017; 8: 20525–20542. 28423528 10.18632/oncotarget.15656 PMC5400524

[44] Zenkov RG, Vlasova OA, Maksimova VP, Fetisov TI, Karpechenko NY, Ektova LV, Molecular Mechanisms of Anticancer Activity of N-Glycosides of Indolocarbazoles LCS-1208 and LCS-1269. Molecules. 2021; 26: 7329. 34885910 10.3390/molecules26237329 PMC8658795

[45] Kanwal R, Datt M, Liu X, Gupta S. Dietary Flavones as Dual Inhibitors of DNA Methyltransferases and Histone Methyltransferases. PLoS ONE. 2016; 11: e0162956. 10.1371/journal.pone.0162956 PMC5033486 27658199

[46] Ji C, Yin X, Duan H, Liang L. Molecular complexes of calf thymus DNA with various bioactive compounds: Formation and characterization. International Journal of Biological Macromolecules. 2021; 168: 775–783. 33227330 10.1016/j.ijbiomac.2020.11.135

[47] Kim TW, Lee HG. Apigenin Induces Autophagy and Cell Death by Targeting EZH2 under Hypoxia Conditions in Gastric Cancer Cells. International Journal of Molecular Sciences. 2021; 22: 13455. 34948250 10.3390/ijms222413455 PMC8706813

[48] Borsoi FT, Neri-Numa IA, de Oliveira WQ, de Araújo FF, Pastore GM. Dietary polyphenols and their relationship to the modulation of non-communicable chronic diseases and epigenetic mechanisms: A mini-review. Food Chemistry. Molecular Sciences. 2022; 6: 100155. 10.1016/j.fochms.2022.100155 PMC9793217 36582744

[49] Li XL, Hu YJ, Wang H, Yu BQ, Yue HL. Molecular spectroscopy evidence of berberine binding to DNA: comparative binding and thermodynamic profile of intercalation. Biomacromolecules. 2012; 13: 873–880. 22316074 10.1021/bm2017959

[50] Mazzini S, Bellucci MC, Mondelli R. Mode of binding of the cytotoxic alkaloid berberine with the double helix oligonucleotide d(AAGAATTCTT)(2). Bioorganic & Medicinal Chemistry. 2003; 11: 505–514. 12538015 10.1016/s0968-0896(02)00466-2

[51] Kandasamy S, Selvaraj M, Muthusamy K, Varadaraju N, Kannupal S, Sekar AK, Structural exploration of common pharmacophore based berberine derivatives as novel histone deacetylase inhibitor targeting HDACs enzymes. Journal of Biomolecular Structure & Dynamics. 2023; 41: 1690–1703. 34994284 10.1080/07391102.2021.2024254

[52] Qing Y, Hu H, Liu Y, Feng T, Meng W, Jiang L, Berberine induces apoptosis in human multiple myeloma cell line U266 through hypomethylation of p53 promoter. Cell Biology International. 2014; 38: 563–570. 24843889 10.1002/cbin.10206

[53] Rehman SU, Sarwar T, Husain MA, Ishqi HM, Tabish M. Studying non-covalent drug-DNA interactions. Archives of Biochemistry and Biophysics. 2015; 576: 49–60. 25951786 10.1016/j.abb.2015.03.024

[54] Sarwar T, Rehman SU, Husain MA, Ishqi HM, Tabish M. Interaction of coumarin with calf thymus DNA: deciphering the mode of binding by in vitro studies. International Journal of Biological Macromolecules. 2015; 73: 9–16. 25453293 10.1016/j.ijbiomac.2014.10.017

[55] Haris P, Mary V, Aparna P, Dileep KV, Sudarsanakumar C. A comprehensive approach to ascertain the binding mode of curcumin with DNA. Spectrochimica Acta. Part A, Molecular and Biomolecular Spectroscopy. 2017; 175: 155–163. 28033562 10.1016/j.saa.2016.11.049

[56] Hahn P. Nutrition and metabolic development. Canadian Journal of Physiology and Pharmacology. 1985; 63: 525–526. 4041996 10.1139/y85-091

[57] Rajendran P, Abdelsalam SA, Renu K, Veeraraghavan V, Ben Ammar R, Ahmed EA. Polyphenols as Potent Epigenetics Agents for Cancer. International Journal of Molecular Sciences. 2022; 23: 11712. 36233012 10.3390/ijms231911712 PMC9570183

[58] Carlos-Reyes Á, López-González JS, Meneses-Flores M, Gallardo-Rincón D, Ruíz-García E, Marchat LA, Dietary Compounds as Epigenetic Modulating Agents in Cancer. Frontiers in Genetics. 2019; 10: 79. 30881375 10.3389/fgene.2019.00079 PMC6406035

[59] Jeong MH, Ko H, Jeon H, Sung GJ, Park SY, Jun WJ, Delphinidin induces apoptosis via cleaved HDAC3-mediated p53 acetylation and oligomerization in prostate cancer cells. Oncotarget. 2016; 7: 56767–56780. 27462923 10.18632/oncotarget.10790 PMC5302952

[60] Ko H, Jeong MH, Jeon H, Sung GJ, So Y, Kim I, Delphinidin sensitizes prostate cancer cells to TRAIL-induced apoptosis, by inducing DR5 and causing caspase-mediated HDAC3 cleavage. Oncotarget. 2015; 6: 9970–9984. 25991668 10.18632/oncotarget.3667 PMC4496411

[61] Kuo HCD, Wu R, Li S, Yang AY, Kong AN. Anthocyanin Delphinidin Prevents Neoplastic Transformation of Mouse Skin JB6 P+ Cells: Epigenetic Re-activation of Nrf2-ARE Pathway. The AAPS Journal. 2019; 21: 83. 31254216 10.1208/s12248-019-0355-5 PMC6669902

[62] Zheng X, Chen A, Hoshi T, Anzai JI, Li G. Electrochemical studies of (−)-epigallocatechin gallate and its interaction with DNA. Analytical and Bioanalytical Chemistry. 2006; 386: 1913–1919. 17019576 10.1007/s00216-006-0752-3

[63] Ghosh KS, Sahoo BK, Jana D, Dasgupta S. Studies on the interaction of copper complexes of (−)-epicatechin gallate and (−)-epigallocatechin gallate with calf thymus DNA. Journal of Inorganic Biochemistry. 2008; 102: 1711–1718. 18541305 10.1016/j.jinorgbio.2008.04.008

[64] Thakur VS, Gupta K, Gupta S. Green tea polyphenols causes cell cycle arrest and apoptosis in prostate cancer cells by suppressing class I histone deacetylases. Carcinogenesis. 2012; 33: 377–384. 22114073 10.1093/carcin/bgr277 PMC3499108

[65] Khan MA, Hussain A, Sundaram MK, Alalami U, Gunasekera D, Ramesh L, (−)-Epigallocatechin-3-gallate reverses the expression of various tumor-suppressor genes by inhibiting DNA methyltransferases and histone deacetylases in human cervical cancer cells. Oncology Reports. 2015; 33: 1976–1984. 25682960 10.3892/or.2015.3802

[66] Ciesielski O, Biesiekierska M, Balcerczyk A. Epigallocatechin-3-gallate (EGCG) Alters Histone Acetylation and Methylation and Impacts Chromatin Architecture Profile in Human Endothelial Cells. Molecules. 2020; 25: 2326. 32429384 10.3390/molecules25102326 PMC7287656

[67] Thakur VS, Gupta K, Gupta S. Green tea polyphenols increase p53 transcriptional activity and acetylation by suppressing class I histone deacetylases. International Journal of Oncology. 2012; 41: 353–361. 22552582 10.3892/ijo.2012.1449 PMC3580388

[68] Sengupta B, Pahari B, Blackmon L, Sengupta PK. Prospect of bioflavonoid fisetin as a quadruplex DNA ligand: a biophysical approach. PloS One. 2013; 8: e65383. 10.1371/journal.pone.0065383 PMC3681855 23785423

[69] Kim A, Yun JM. Combination Treatments with Luteolin and Fisetin Enhance Anti-Inflammatory Effects in High Glucose-Treated THP-1 Cells Through Histone Acetyltransferase/Histone Deacetylase Regulation. Journal of Medicinal Food. 2017; 20: 782–789. 28650731 10.1089/jmf.2017.3968

[70] Ding G, Xu X, Li D, Chen Y, Wang W, Ping D, Fisetin inhibits proliferation of pancreatic adenocarcinoma by inducing DNA damage via RFXAP/KDM4A-dependent histone H3K36 demethylation. Cell Death & Disease. 2020; 11: 893. 33093461 10.1038/s41419-020-03019-2 PMC7582166

[71] Sundaram MK, Unni S, Somvanshi P, Bhardwaj T, Mandal RK, Hussain A, Genistein Modulates Signaling Pathways and Targets Several Epigenetic Markers in HeLa Cells. Genes. 2019; 10: 955. 31766427 10.3390/genes10120955 PMC6947182

[72] Ma L, Liu S, Xu N-S, Jiang Y-Q, Song F-R and Liu Z-Q. Interactions of ginsenosides with DNA duplexes: A study by electrospray ionization mass spectrometry and UV absorption spectroscopy. Chinese Chemical Letters. 2014; 25: 1179–1184.

[73] Shan X, Fu YS, Aziz F, Wang XQ, Yan Q, Liu JW. Ginsenoside Rg3 inhibits melanoma cell proliferation through downregulation of histone deacetylase 3 (HDAC3) and increase of p53 acetylation. PLoS ONE. 2014; 9: e115401. 10.1371/journal.pone.0115401 PMC4270766 25521755

[74] Kanakis CD, Tarantilis PA, Polissiou MG, Diamantoglou S, Tajmir-Riahi HA. DNA interaction with naturally occurring antioxidant flavonoids quercetin, kaempferol, and delphinidin. Journal of Biomolecular Structure & Dynamics. 2005; 22: 719–724. 15842176 10.1080/07391102.2005.10507038

[75] Kim TW, Lee SY, Kim M, Cheon C, Ko SG. Kaempferol induces autophagic cell death via IRE1-JNK-CHOP pathway and inhibition of G9a in gastric cancer cells. Cell Death & Disease. 2018; 9: 875. 30158521 10.1038/s41419-018-0930-1 PMC6115440

[76] Qiu W, Lin J, Zhu Y, Zhang J, Zeng L, Su M, Kaempferol Modulates DNA Methylation and Downregulates DNMT3B in Bladder Cancer. Cellular Physiology and Biochemistry. 2017; 41: 1325–1335. 28278502 10.1159/000464435

[77] Imran M, Salehi B, Sharifi-Rad J, Aslam Gondal T, Saeed F, Imran A, Kaempferol: A Key Emphasis to Its Anticancer Potential. Molecules. 2019; 24: 2277. 31248102 10.3390/molecules24122277 PMC6631472

[78] Prakash O, Singh R, Singh N, Usmani S, Arif M, Kumar R, Anticancer Potential of Naringenin, Biosynthesis, Molecular Target, and Structural Perspectives. Mini Reviews in Medicinal Chemistry. 2022; 22: 758–769. 34517796 10.2174/1389557521666210913112733

[79] Wen C, Lu X, Sun Y, Li Q, Liao J, Li L. Naringenin induces the cell apoptosis of acute myeloid leukemia cells by regulating the lncRNA XIST/miR-34a/HDAC1 signaling. Heliyon. 2023; 9: e15826. 10.1016/j.heliyon.2023.e15826 PMC10189189 37206002

[80] Baranowska M, Koziara Z, Suliborska K, Chrzanowski W, Wormstone M, Namieśnik J, Interactions between polyphenolic antioxidants quercetin and naringenin dictate the distinctive redox-related chemical and biological behaviour of their mixtures. Scientific Reports. 2021; 11: 12282. 34112813 10.1038/s41598-021-89314-0 PMC8192515

[81] Mitrasinovic PM. Sequence-dependent binding of flavonoids to duplex DNA. Journal of Chemical Information and Modeling. 2015; 55: 421–433. 25580618 10.1021/ci5006965

[82] Kedhari Sundaram M, Hussain A, Haque S, Raina R, Afroze N. Quercetin modifies 5’CpG promoter methylation and reactivates various tumor suppressor genes by modulating epigenetic marks in human cervical cancer cells. Journal of Cellular Biochemistry. 2019; 120: 18357–18369. 31172592 10.1002/jcb.29147

[83] Zhang S, Sun X, Jing Z, Qu F. Spectroscopic analysis on the resveratrol-DNA binding interactions at physiological pH. Spectrochimica Acta. Part A, Molecular and Biomolecular Spectroscopy. 2011; 82: 213–216. 21856217 10.1016/j.saa.2011.07.037

[84] Fernandes GFS, Silva GDB, Pavan AR, Chiba DE, Chin CM, Dos Santos JL. Epigenetic Regulatory Mechanisms Induced by Resveratrol. Nutrients. 2017; 9: 1201. 29104258 10.3390/nu9111201 PMC5707673

[85] Banerjee A, Singh J, Dasgupta D. Fluorescence spectroscopic and calorimetry based approaches to characterize the mode of interaction of small molecules with DNA. Journal of Fluorescence. 2013; 23: 745–752. 23494169 10.1007/s10895-013-1211-0

[86] Selvi BR, Pradhan SK, Shandilya J, Das C, Sailaja BS, Shankar GN, Sanguinarine interacts with chromatin, modulates epigenetic modifications, and transcription in the context of chromatin. Chemistry & Biology. 2009; 16: 203–216. 19246011 10.1016/j.chembiol.2008.12.006

[87] Parvin SI, Mandal MK, Gopi P, Singh S, Khan MR, Pandya P, A comparative study on DNA and protein binding properties of thymol and thymoquinone. Journal of Biomolecular Structure & Dynamics. 2023; 41: 10944–10956. 36841618 10.1080/07391102.2023.2180665

[88] Khan MA, Tania M, Fu J. Epigenetic role of thymoquinone: impact on cellular mechanism and cancer therapeutics. Drug Discovery Today. 2019; 24: 2315–2322. 31541714 10.1016/j.drudis.2019.09.007

[89] Parbin S, Shilpi A, Kar S, Pradhan N, Sengupta D, Deb M, Insights into the molecular interactions of thymoquinone with histone deacetylase: evaluation of the therapeutic intervention potential against breast cancer. Molecular BioSystems. 2016; 12: 48–58. 26540192 10.1039/c5mb00412h

[90] Fitz-James MH, Cavalli G. Molecular mechanisms of trans-generational epigenetic inheritance. Nature Reviews. Genetics. 2022; 23: 325–341. 10.1038/s41576-021-00438-5 PMC7619059 34983971

[91] Spadafora C The epigenetic basis of evolution. Progress in Biophysics and Molecular Biology. 2023; 178: 57–69. 36720315 10.1016/j.pbiomolbio.2023.01.005

[92] Maksimova V, Popova V, Prus A, Lylova E, Usalka O, Sagitova G, Insights into the Mechanism of Curaxin CBL0137 Epigenetic Activity: The Induction of DNA Demethylation and the Suppression of BET Family Proteins. International Journal of Molecular Sciences. 2023; 24: 12874. 37629054 10.3390/ijms241612874 PMC10454690

[93] Rabbani-Chadegani A, Mollaei H, Sargolzaei J. Investigation of the interaction between berberine and nucleosomes in solution: Spectroscopic and equilibrium dialysis approach. Spectrochimica Acta. Part A, Molecular and Biomolecular Spectroscopy. 2017; 173: 418–424. 27705846 10.1016/j.saa.2016.09.052

[94] Kantidze OL, Luzhin AV, Nizovtseva EV, Safina A, Valieva ME, Golov AK, The anti-cancer drugs curaxins target spatial genome organization. Nature Communications. 2019; 10: 1441. 10.1038/s41467-019-09500-7 PMC6441033 30926878

[95] Volokh OI, Sivkina AL, Moiseenko AV, Popinako AV, Karlova MG, Valieva ME, Mechanism of curaxin-dependent nucleosome unfolding by FACT. Frontiers in Molecular Biosciences. 2022; 9: 1048117. 10.3389/fmolb.2022.1048117 PMC9723464 36483541

[96] Fan Y, Nikitina T, Zhao J, Fleury TJ, Bhattacharyya R, Bouhassira EE, Histone H1 depletion in mammals alters global chromatin structure but causes specific changes in gene regulation. Cell. 2005; 123: 1199–1212. 16377562 10.1016/j.cell.2005.10.028

[97] Kumar A, Maurya P, Hayes JJ. Post-Translation Modifications and Mutations of Human Linker Histone Subtypes: Their Manifestation in Disease. International Journal of Molecular Sciences. 2023; 24: 1463. 36674981 10.3390/ijms24021463 PMC9860689

[98] Chubb JE, Rea S. Core and linker histone modifications involved in the DNA damage response. Sub-cellular Biochemistry. 2010; 50: 17–42. 20012575 10.1007/978-90-481-3471-7_2

[99] Fernández-Justel JM, Santa-María C, Martín-Vírgala S, Ramesh S, Ferrera-Lagoa A, Salinas-Pena M, Histone H1 regulates non-coding RNA turnover on chromatin in a m6A-dependent manner. Cell Reports. 2022; 40: 111329. 10.1016/j.celrep.2022.111329 PMC7613722 36103831

[100] Pan C, Fan Y. Role of H1 linker histones in mammalian development and stem cell differentiation. Biochimica et Biophysica Acta. 2016; 1859: 496–509. 26689747 10.1016/j.bbagrm.2015.12.002 PMC4775330

[101] Scaffidi P Histone H1 alterations in cancer. Biochimica et Biophysica Acta. 2016; 1859: 533–539. 26386351 10.1016/j.bbagrm.2015.09.008

[102] Hechtman JF, Beasley MB, Kinoshita Y, Ko HM, Hao K, Burstein DE. Promyelocytic leukemia zinc finger and histone H1.5 differentially stain low- and high-grade pulmonary neuroendocrine tumors: a pilot immunohistochemical study. Human Pathology. 2013; 44: 1400–1405. 23416030 10.1016/j.humpath.2012.11.014

[103] Khachaturov V, Xiao GQ, Kinoshita Y, Unger PD, Burstein DE. Histone H1.5, a novel prostatic cancer marker: an immunohistochemical study. Human Pathology. 2014; 45: 2115–2119. 25130394 10.1016/j.humpath.2014.06.015

[104] Kostova NN, Srebreva LN, Milev AD, Bogdanova OG, Rundquist I, Lindner HH, Immunohistochemical demonstration of histone H1(0) in human breast carcinoma. Histochemistry and Cell Biology. 2005; 124: 435–443. 16158288 10.1007/s00418-005-0052-6

[105] Torres CM, Biran A, Burney MJ, Patel H, Henser-Brownhill T, Cohen AHS, The linker histone H1.0 generates epigenetic and functional intratumor heterogeneity. Science. 2016; 353: aaf1644. 10.1126/science.aaf1644 PMC5131846 27708074

[106] Medrzycki M, Zhang Y, McDonald JF, Fan Y. Profiling of linker histone variants in ovarian cancer. Frontiers in Bioscience (Landmark Edition). 2012; 17: 396–406. 22201751 10.2741/3934 PMC3754803

[107] Noberini R, Morales Torres C, Savoia EO, Brandini S, Jodice MG, Bertalot G, Label-Free Mass Spectrometry-Based Quantification of Linker Histone H1 Variants in Clinical Samples. International Journal of Molecular Sciences. 2020; 21: 7330. 33020374 10.3390/ijms21197330 PMC7582528

[108] Zhou S, Yan Y, Chen X, Zeng S, Wei J, Wang X, A two-gene-based prognostic signature for pancreatic cancer. Aging. 2020; 12: 18322–18342. 32966237 10.18632/aging.103698 PMC7585105

[109] Cao K, Lailler N, Zhang Y, Kumar A, Uppal K, Liu Z, High-resolution mapping of h1 linker histone variants in embryonic stem cells. PLoS Genetics. 2013; 9: e1003417. 10.1371/journal.pgen.1003417 PMC3636266 23633960

[110] Fan Y, Nikitina T, Morin-Kensicki EM, Zhao J, Magnuson TR, Woodcock CL, H1 linker histones are essential for mouse development and affect nucleosome spacing in vivo. Molecular and Cellular Biology. 2003; 23: 4559–4572. 12808097 10.1128/MCB.23.13.4559-4572.2003 PMC164858

[111] Fan Y, Sirotkin A, Russell RG, Ayala J, Skoultchi AI. Individual somatic H1 subtypes are dispensable for mouse development even in mice lacking the H1(0) replacement subtype. Molecular and Cellular Biology. 2001; 21: 7933–7943. 11689686 10.1128/MCB.21.23.7933-7943.2001 PMC99962

[112] Talasz H, Helliger W, Puschendorf B, Lindner H. In vivo phosphorylation of histone H1 variants during the cell cycle. Biochemistry. 1996; 35: 1761–1767. 8639656 10.1021/bi951914e

[113] Pateetin P, Pisitkun T, McGowan E, Boonyaratanakornkit V. Differential quantitative proteomics reveals key proteins related to phenotypic changes of breast cancer cells expressing progesterone receptor A. The Journal of Steroid Biochemistry and Molecular Biology. 2020; 198: 105560. 10.1016/j.jsbmb.2019.105560 31809870

[114] Ren M, Yang L, Li D, Yang L, Su Y, Su X. Cell Cycle Regulation by Berberine in Human Melanoma A375 Cells. Bulletin of Experimental Biology and Medicine. 2020; 169: 491–496. 32915362 10.1007/s10517-020-04916-4

[115] Samad MA, Saiman MZ, Abdul Majid N, Karsani SA, Yaacob JS. Berberine Inhibits Telomerase Activity and Induces Cell Cycle Arrest and Telomere Erosion in Colorectal Cancer Cell Line, HCT 116. Molecules. 2021; 26: 376. 33450878 10.3390/molecules26020376 PMC7828342

[116] Chou YT, Koh YC, Nagabhushanam K, Ho CT, Pan MH. A Natural Degradant of Curcumin, Feruloylacetone Inhibits Cell Proliferation via Inducing Cell Cycle Arrest and a Mitochondrial Apoptotic Pathway in HCT116 Colon Cancer Cells. Molecules. 2021; 26: 4884. 34443472 10.3390/molecules26164884 PMC8399060

[117] Hesari A, Rezaei M, Rezaei M, Dashtiahangar M, Fathi M, Rad JG, Effect of curcumin on glioblastoma cells. Journal of Cellular Physiology. 2019; 234: 10281–10288. 30585634 10.1002/jcp.27933

[118] Li Y-Y, Liu H-C, Zhang Q, Feng R-T, Song Y-S, Ming L. Effect of Curcumin on the Proliferation, Apoptosis, and Cell Cycle of Human Acute Myeloid Leukemia Cell Line K562. Zhongguo Shi Yan Xue Ye Xue Za Zhi. 2022; 30: 1343–1347. (In Chinese) 36208233 10.19746/j.cnki.issn.1009-2137.2022.05.007

[119] Ye Z, Chen D, Zheng R, Chen H, Xu T, Wang C, Curcumin induced G2/M cycle arrest in SK-N-SH neuroblastoma cells through the ROS-mediated p53 signaling pathway. Journal of Food Biochemistry. 2021; 45: e13888. 10.1111/jfbc.13888 34331475

[120] Qi Z, Kong S, Zhao S, Tang Q. Naringenin inhibits human breast cancer cells (MDA-MB-231) by inducing programmed cell death, caspase stimulation, G2/M phase cell cycle arrest and suppresses cancer metastasis. Cellular and Molecular Biology. 2021; 67: 8–13. 34817344 10.14715/cmb/2021.67.2.2

[121] Serna-Pujol N, Salinas-Pena M, Mugianesi F, Le Dily F, Marti-Renom MA, Jordan A. Coordinated changes in gene expression, H1 variant distribution and genome 3D conformation in response to H1 depletion. Nucleic Acids Research. 2022; 50: 3892–3910. 35380694 10.1093/nar/gkac226 PMC9023279

[122] Healton SE, Pinto HD, Mishra LN, Hamilton GA, Wheat JC, Swist-Rosowska K, H1 linker histones silence repetitive elements by promoting both histone H3K9 methylation and chromatin compaction. Proceedings of the National Academy of Sciences of the United States of America. 2020; 117: 14251–14258. 32513732 10.1073/pnas.1920725117 PMC7322038

[123] Hansen JC. Silencing the genome with linker histones. Proceedings of the National Academy of Sciences of the United States of America. 2020; 117: 15388–15390. 32561644 10.1073/pnas.2009513117 PMC7355042

[124] Mu X, Ahmad S, Hur S. Endogenous Retroelements and the Host Innate Immune Sensors. Advances in Immunology. 2016; 132: 47–69. 27769507 10.1016/bs.ai.2016.07.001 PMC5135014

[125] Roers A, Hiller B, Hornung V. Recognition of Endogenous Nucleic Acids by the Innate Immune System. Immunity. 2016; 44: 739–754. 27096317 10.1016/j.immuni.2016.04.002

[126] Gozgit JM, Vasbinder MM, Abo RP, Kunii K, Kuplast-Barr KG, Gui B, PARP7 negatively regulates the type I interferon response in cancer cells and its inhibition triggers antitumor immunity. Cancer Cell. 2021; 39: 1214–1226.e10. 34375612 10.1016/j.ccell.2021.06.018

