# Supplementary material for: Anticancer Plant Secondary Metabolites Induce Linker Histone Depletion from Chromatin
Source: Front Biosci (Landmark Ed). Author manuscript; Available in PMC 2025 Dec 16. (PMC12707778; doi:10.31083/j.fbl2908275)
Supplement: Supplementary Materials [file NIHMS2117097-supplement-Supplementary_Materials.docx]

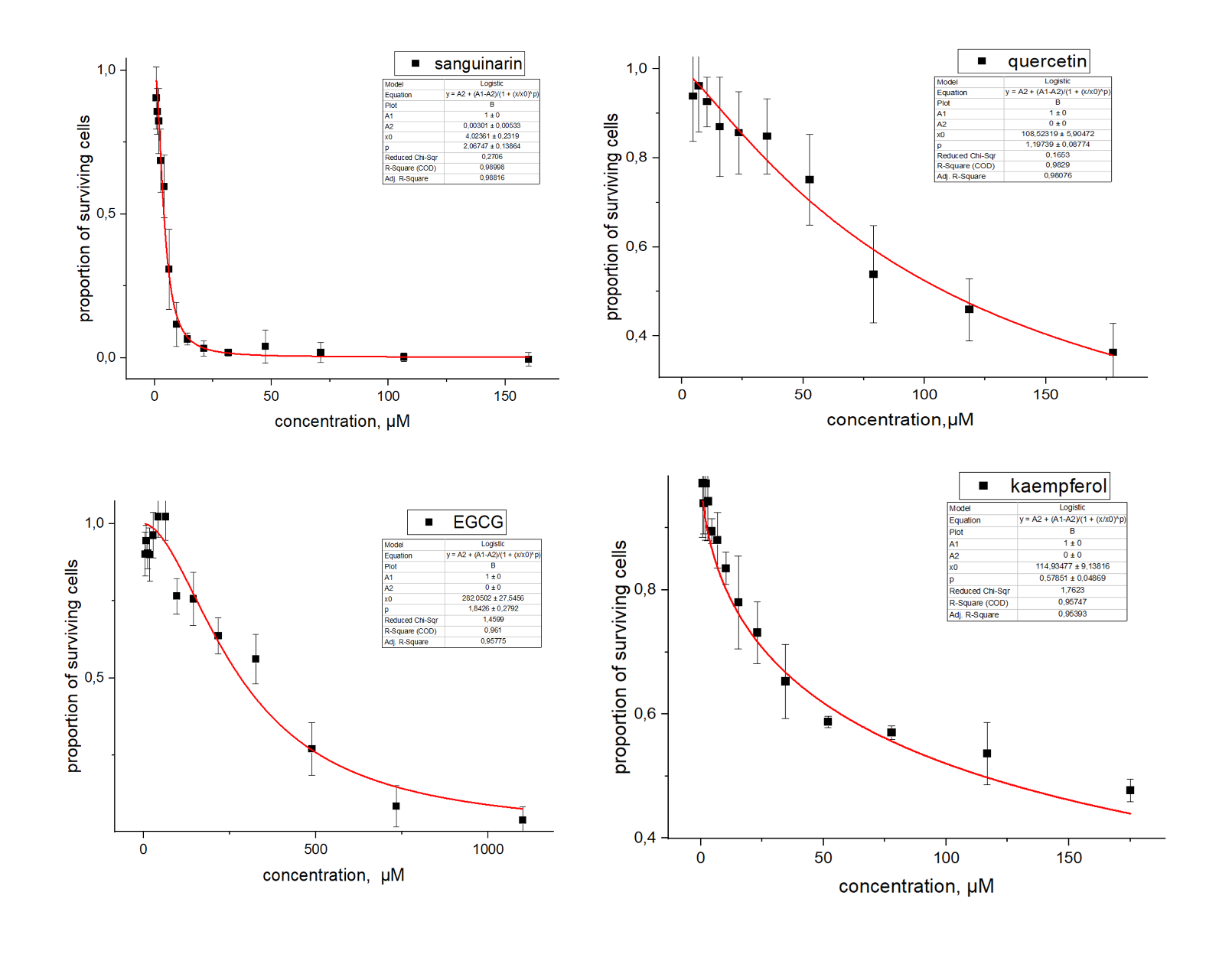


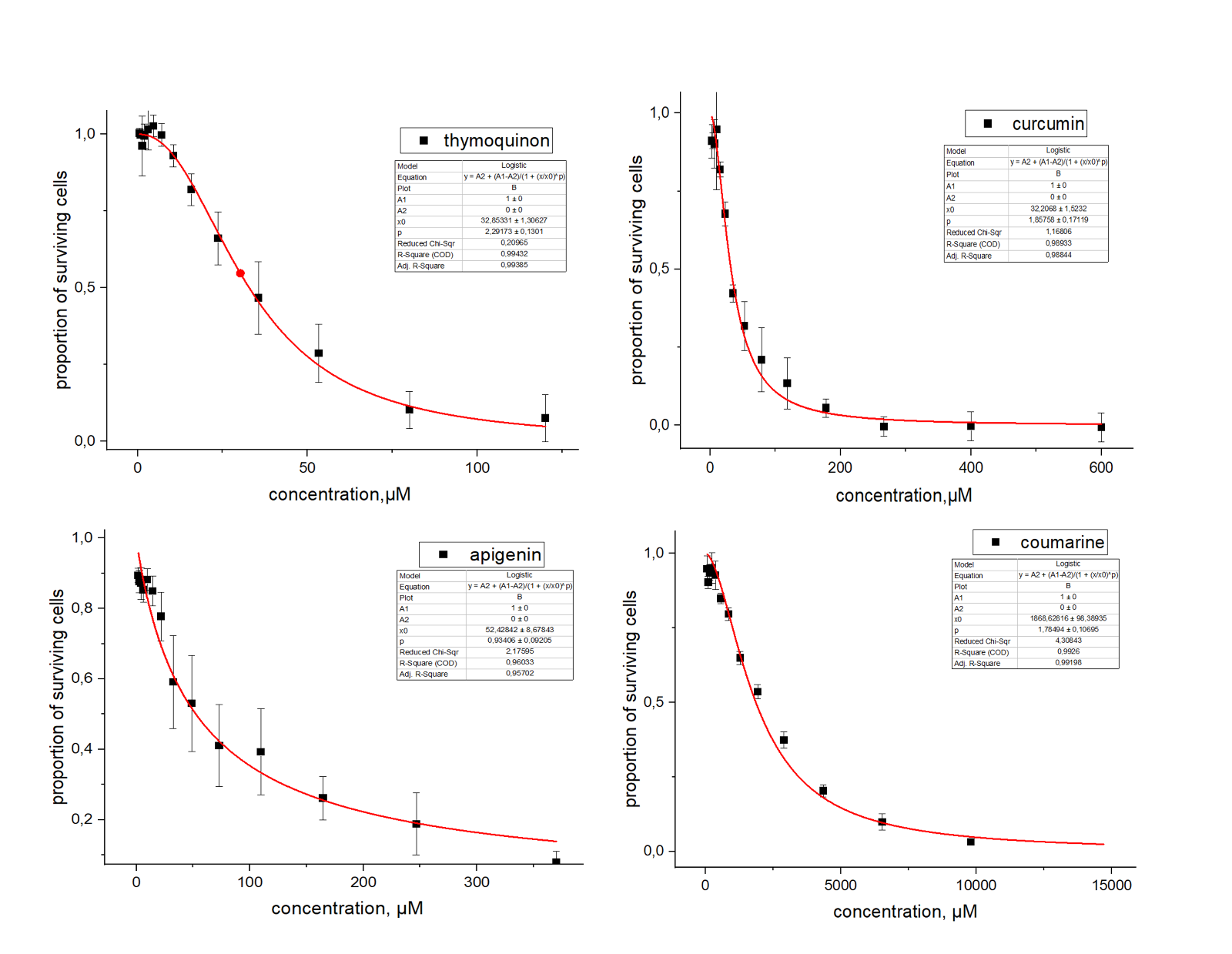


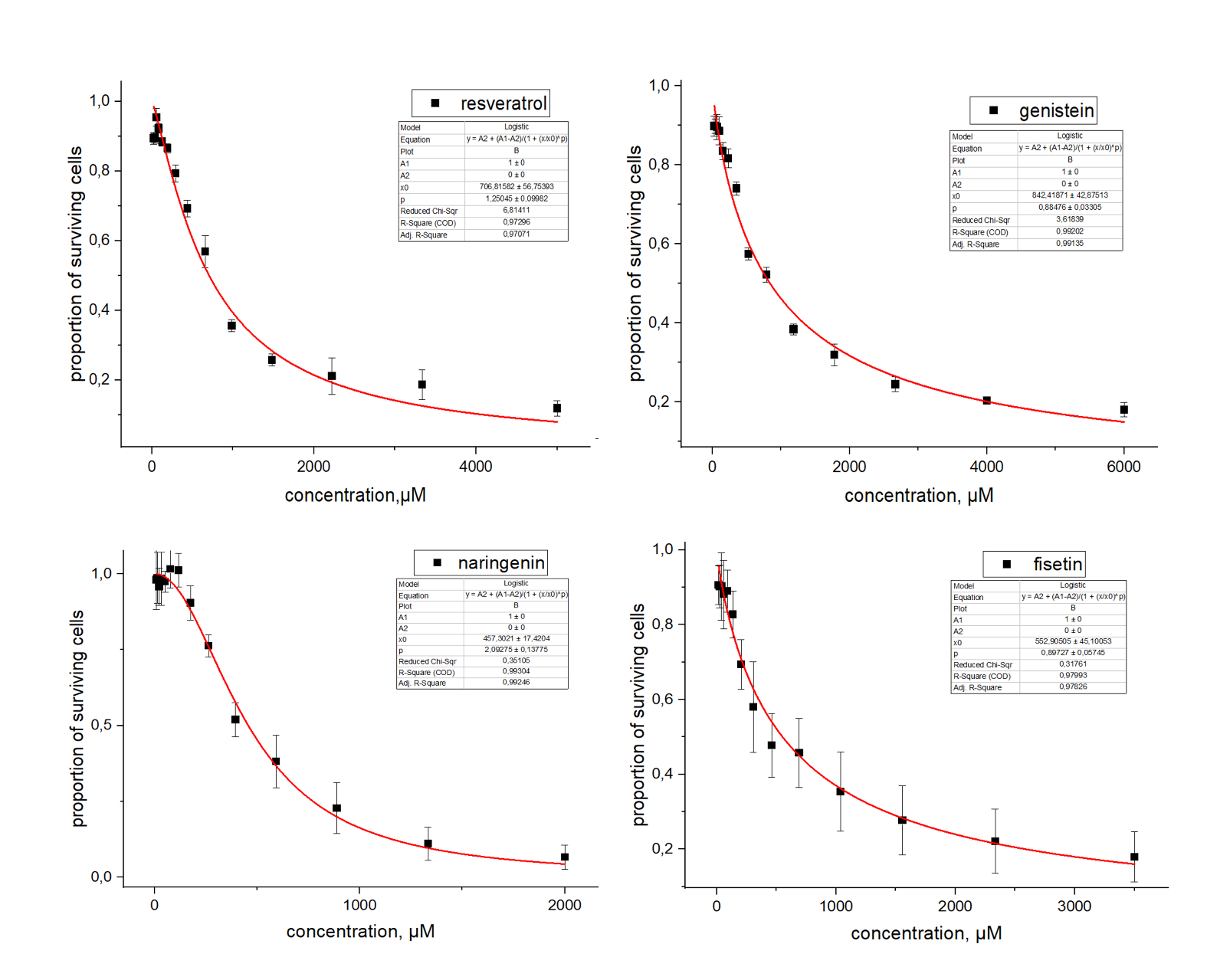


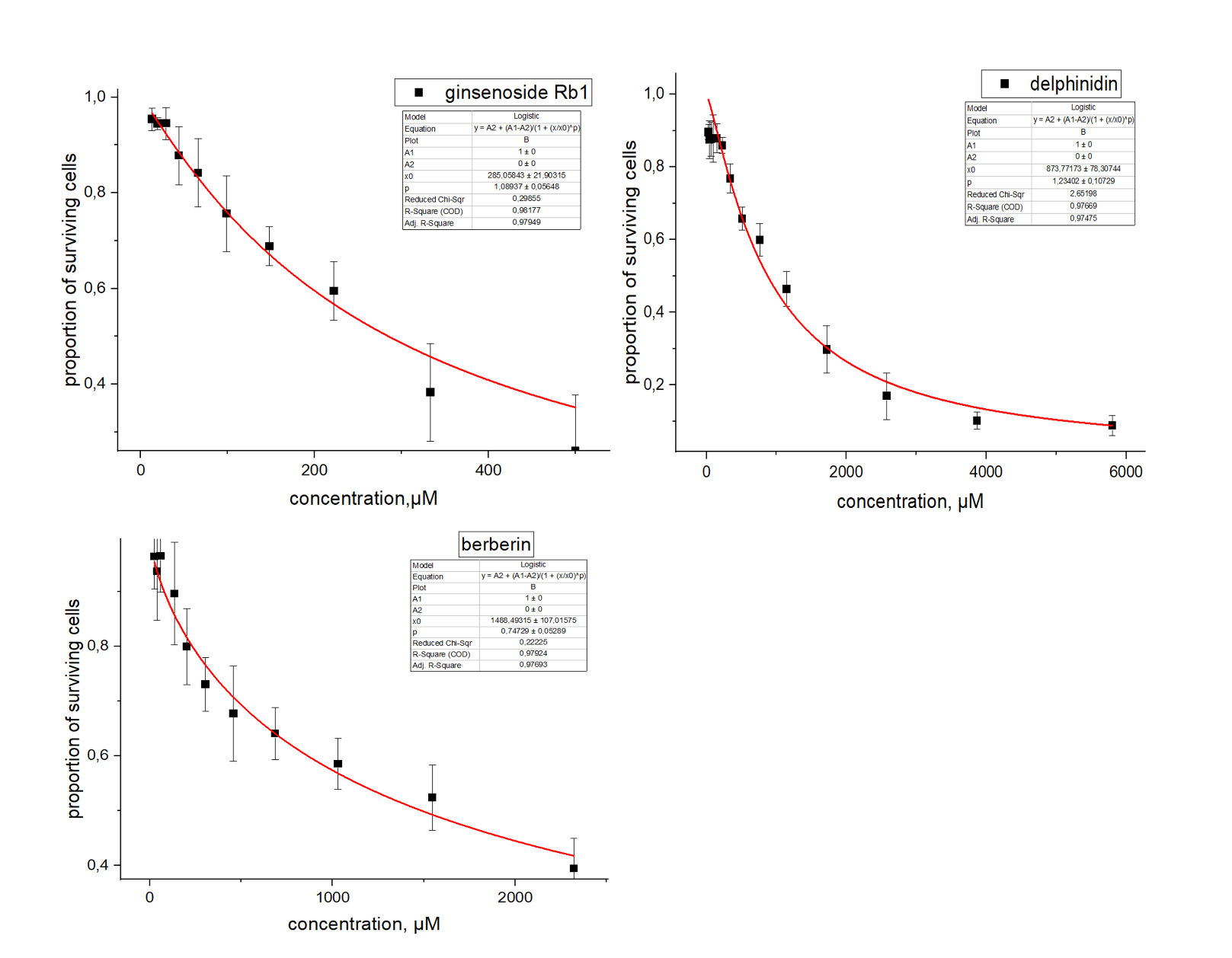


Supplementary Fig. 1. Aproximate logistic function of curves resorufin fluorescence of plant secondary metabolites.


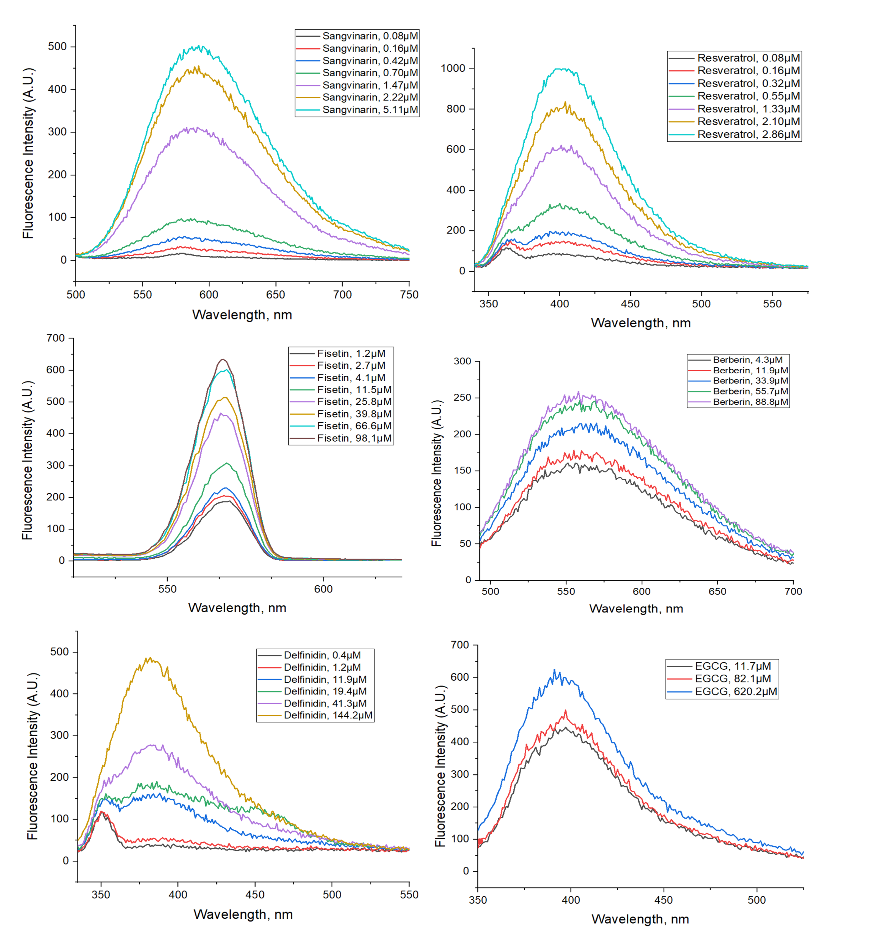


Supplementary Fig. 2. Fluorescence spectra of the PSMs.


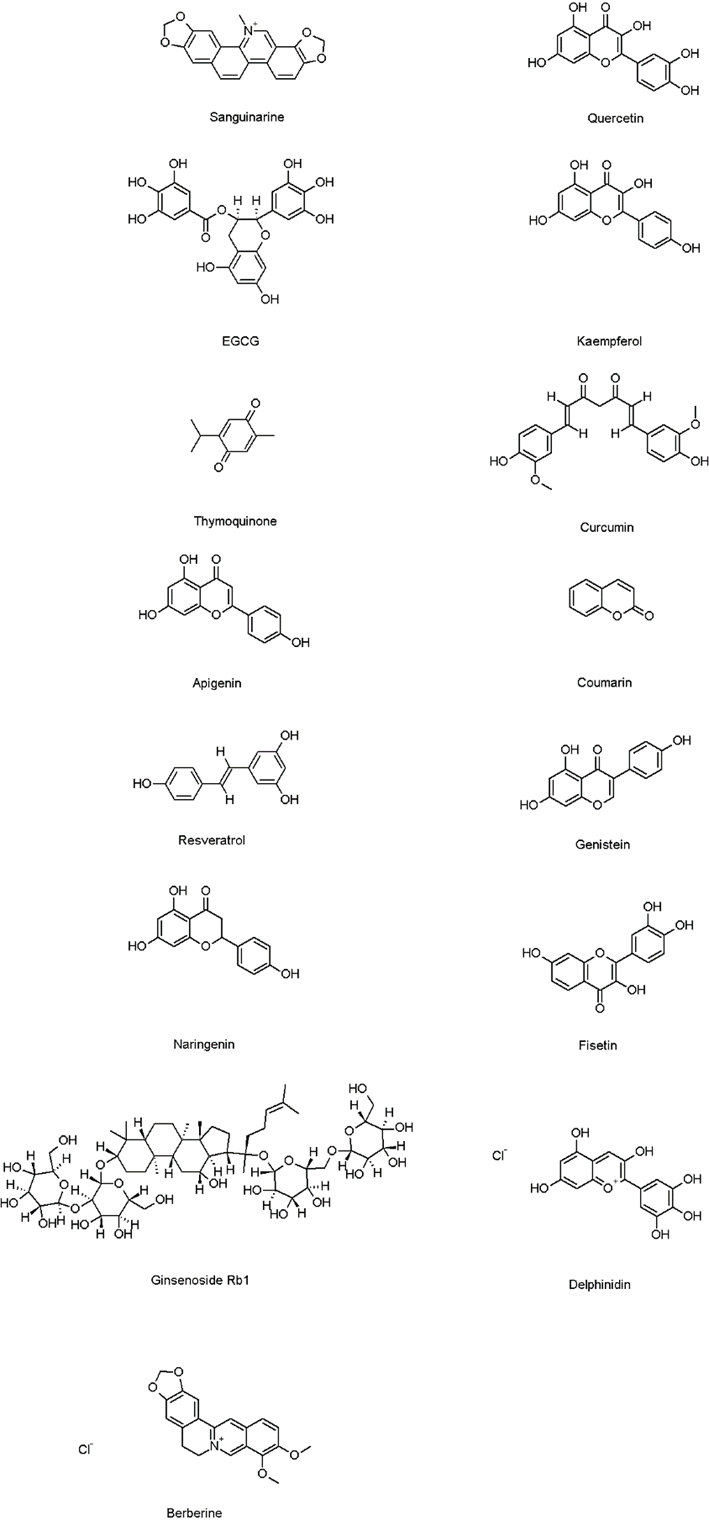


Supplementary Fig. 3. Structural formulae of plant secondary metabolites.

A.


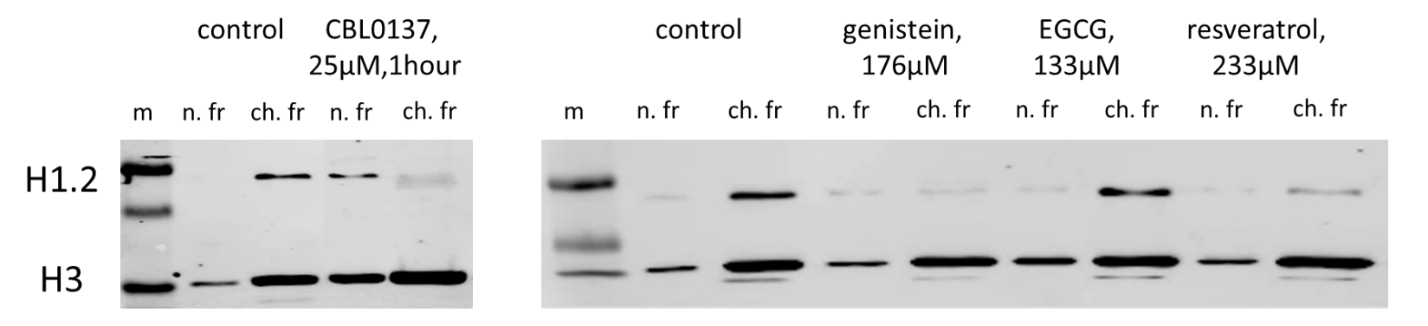


B.


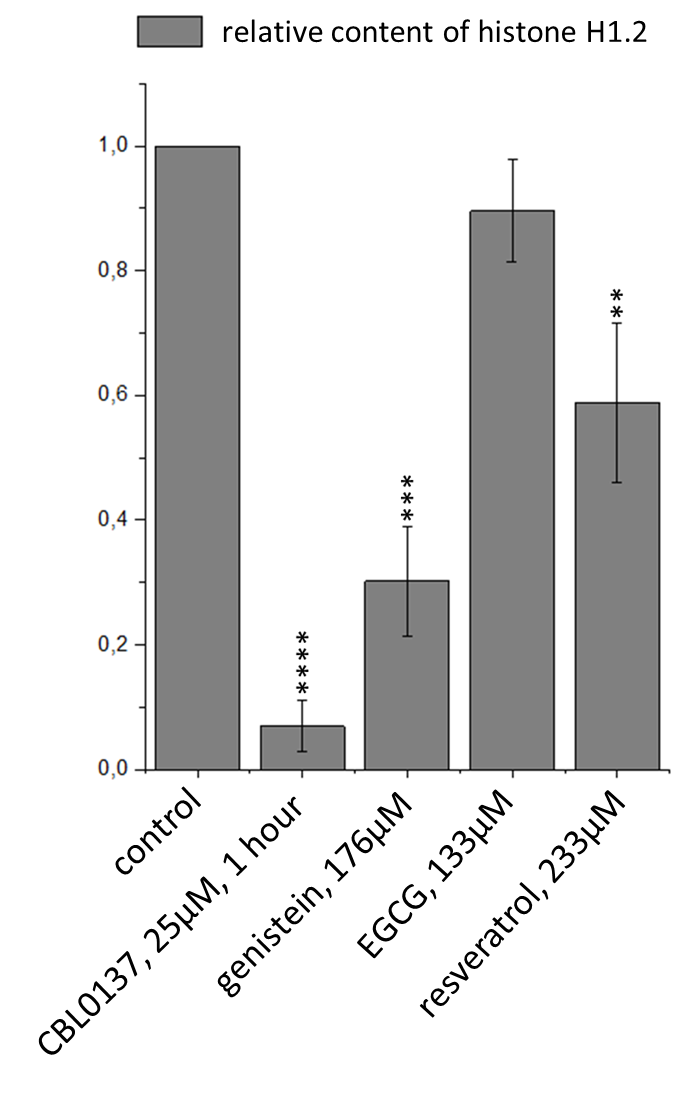


Supplementary Fig. 4.

Depletion of linker histones H1.2 under the treatment of T47D cells with CBL0137 and PSMs. A. Western blotting analysis of the nucleoplasmic and chromatin fractions of T47D cells treated with PSMs for 24h. (m-protein marker, n. fr- nucleoplasmic fraction, ch. fr- chromatin fraction). B. Densitometry analysis of the blots: Mean ± SD; statistically significance of the differences of the relative histone contents (ANOVA test and Dunnett’s post hoc test): ** – p<0.01, *** – p<0.001, **** – p<0.0001.


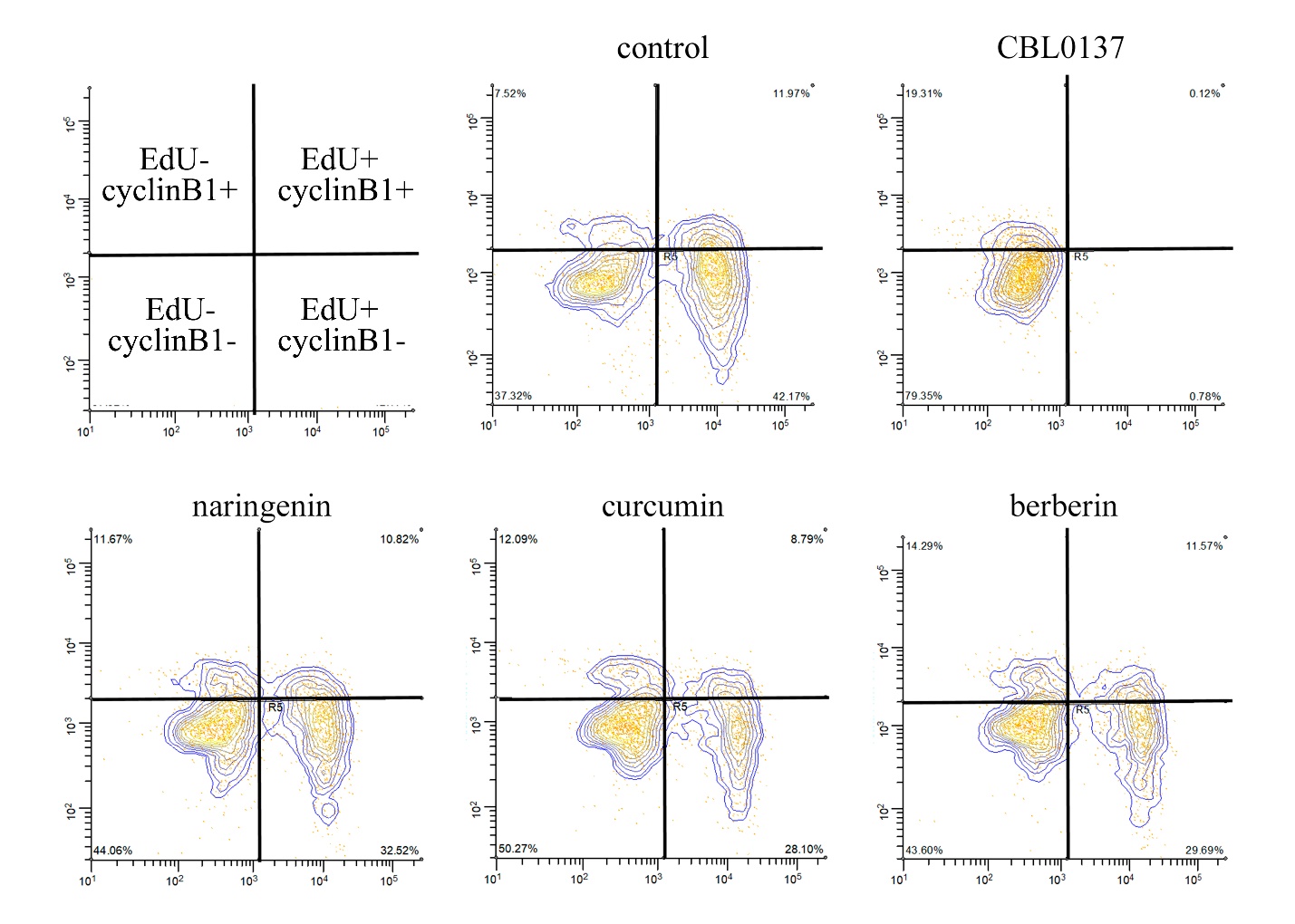


Supplementary Fig. 5.

Flow cytometry results by EdU and cyclin B1 markers for cell cycle distributions detected in populations of untreated cells and cells treated for 1 hour with 25 µM CBL0137, and with berberin, curcumin, and naringenin at the highest non-toxic concentrations.


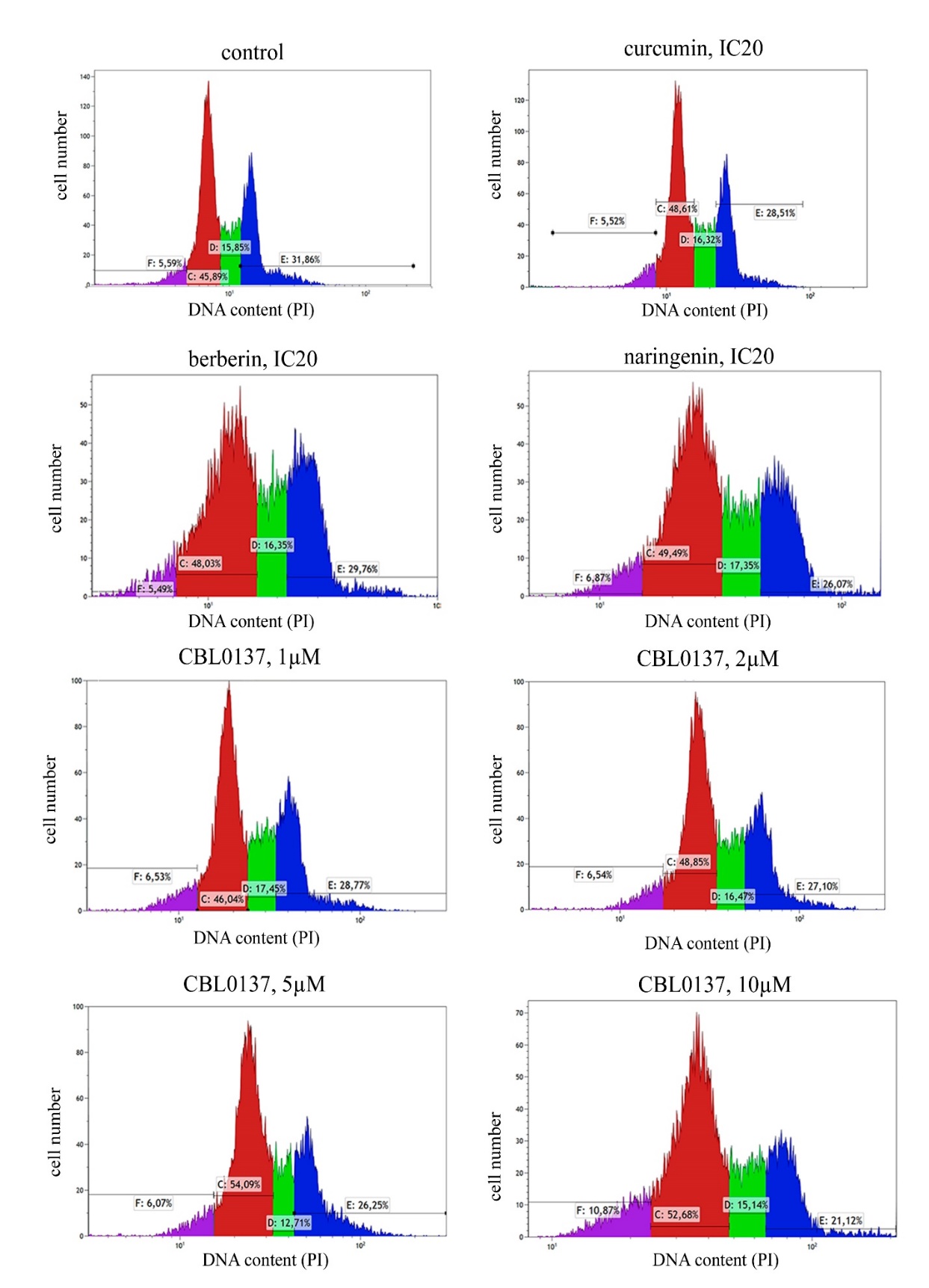


Supplementary Fig. 6

Flow cytometry results by PI marker for cell cycle distributions detected in populations of untreated cells and cells treated for 1 hour with CBL0137, and with berberin, curcumin, and naringenin at the IC20 concentrations


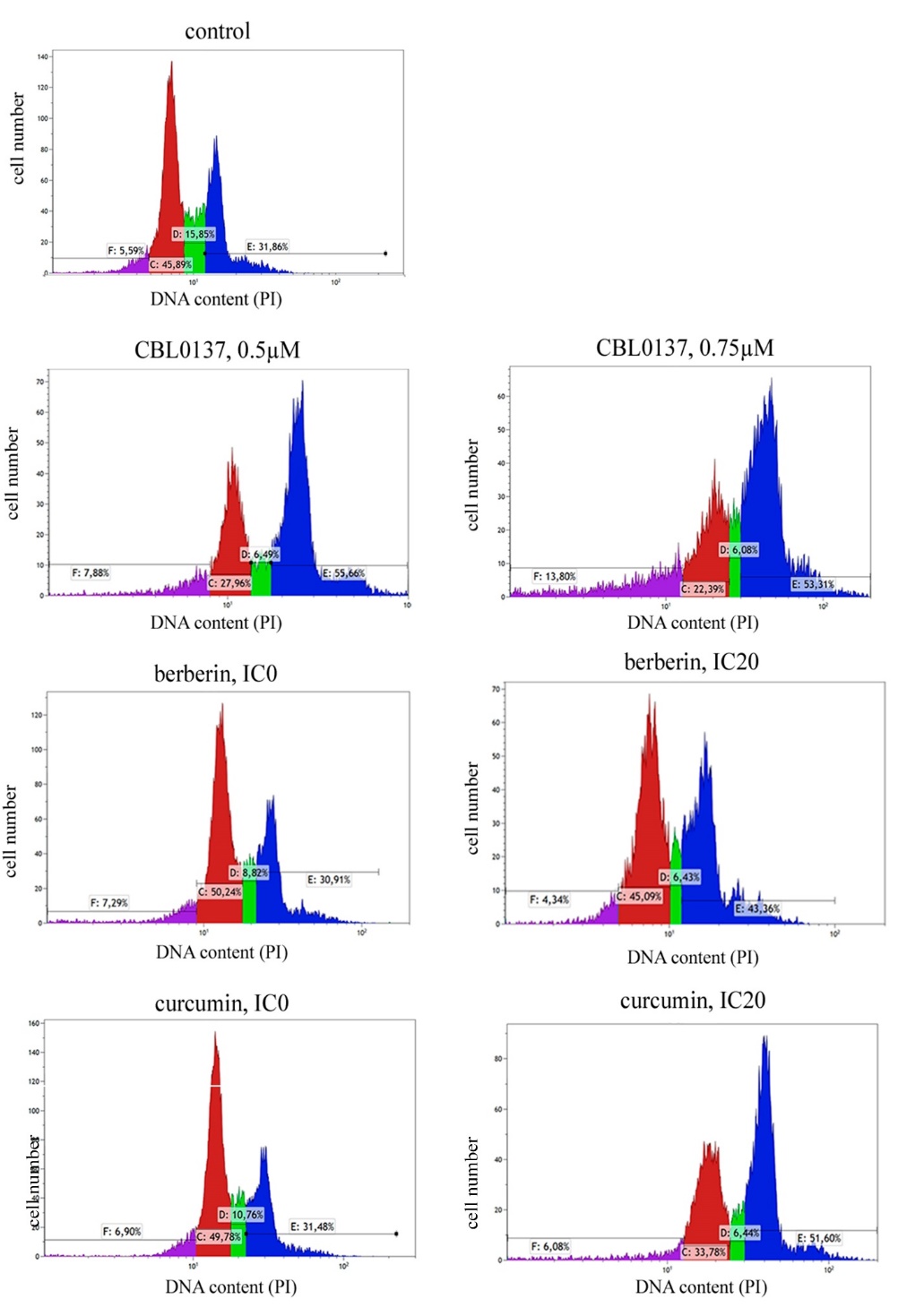


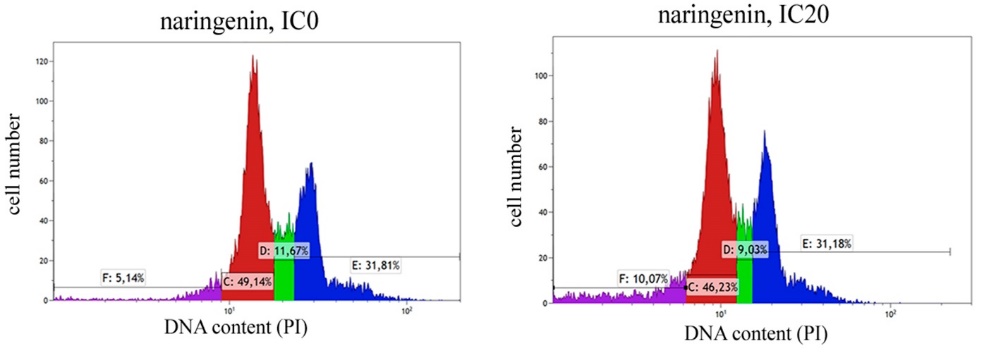


Supplementary Fig. 7

Flow cytometry results by PI marker for cell cycle distributions detected in populations of untreated cells and cells treated for 24 hours with CBL0137, and with berberin, curcumin, and naringenin at the IC20 concentrations and at the highest non-toxic concentrations

Supplementary Table 1. Fluorescence data of the PSMs.

| PSM | Excitation, nm | Emission, nm | Laser voltage, v | Fluorescence | Effect on fluorescence in experiment | |
| --- | --- | --- | --- | --- | --- | --- |
|  |  |  |  |  | **mCherry** | **AlexaFluor-647** |
| Sanguinarine | 480 | 600 | 900 | High level | At concentration > IC20 |  |
| Resveratrol | 319 | 400 | 900 | High level | no |  |
| Fisetin | 280 | 567 | 900 | High level | At concentration > IC20 |  |
| Berberin | 458 | 550 | 900 | Low level | At concentration > IC50 | no |
| Delphinidin | 310 | 381 | 900 | Low level | no |  |
| EGCG | 328 | 391 | 900 | Low level | no |  |
| Quercetin |  |  |  | no | no |  |
| Kaempferol |  |  |  | no | no |  |
| Thymoquinon |  |  |  | no | no |  |
| Curcumin |  |  |  | no | no | no |
| Apigenin |  |  |  | no | no |  |
| Coumarin |  |  |  | no | no |  |
| Genistein |  |  |  | no | no |  |
| Naringenin |  |  |  | no | no | no |
| Ginsenoside Rb1 |  |  |  | no | no |  |
